# Bayesian material flow analysis for systems with multiple levels of disaggregation and high dimensional data

**DOI:** 10.1111/jiec.13550

**Published:** 2024-09-30

**Authors:** Junyang Wang, Kolyan Ray, Pablo Brito-Parada, Yves Plancherel, Tom Bide, Joseph Mankelow, John Morley, Julia A. Stegemann, Rupert Myers

**Affiliations:** 1 Department of Civil and Environmental Engineering, Imperial College London, London, UK; 2 Department of Earth Science and Engineering, Imperial College London, London, UK; 3 Department of Mathematics, Imperial College London, London, UK; 4 Department of Civil, Environmental and Geomatic Engineering, University College London, London, UK; 5 British Geological Survey, Nottingham, UK

**Keywords:** Bayesian statistics, circular economy, material flow analysis, missing data, probabilistic modeling, uncertainty quantification

## Abstract

Material flow analysis (MFA) is used to quantify and understand the life cycles of materials from production to end of use, which enables environmental, social, and economic impacts and interventions. MFA is challenging as available data are often limited and uncertain, leading to an under-determined system with an infinite number of possible stocks and flows values. Bayesian statistics is an effective way to address these challenges by principally incorporating domain knowledge, quantifying uncertainty in the data, and providing probabilities associated with model solutions. This paper presents a novel MFA methodology under the Bayesian framework. By relaxing the mass balance constraints, we improve the computational scalability and reliability of the posterior samples compared to existing Bayesian MFA methods. We propose a mass-based, child and parent process framework to model systems with disaggregated processes and flows. We show posterior predictive checks can be used to identify inconsistencies in the data and aid noise and hyperparameter selection. The proposed approach is demonstrated in case studies, including a global aluminum cycle with significant disaggregation, under weakly informative priors and significant data gaps to investigate the feasibility of Bayesian MFA. We illustrate that just a weakly informative prior can greatly improve the performance of Bayesian methods, for both estimation accuracy and uncertainty quantification.

**Supplementary Information:**

The online version of this article (doi:10.1111/jiec.13550) contains supplementary material, which is available to authorized users.

## INTRODUCTION

Increasingly, the world is facing major shifts in resource utilization. To prevent the worst consequences of climate change, global carbon emissions must be significantly reduced, which requires a fundamental change in how fossil fuels and other high carbon footprint materials are used in the coming decades. Additionally, the instability of supply chains, changing behavior caused by COVID-19 (Jowitt, 2020), as well as the growing world population, projected to reach almost 10 billion by 2050 (WPP, 2022), will also likely significantly impact resource utilization globally (Jowitt et al., 2020). However, many material flows of interest are poorly understood, which severely impedes the ability of policy-makers to identify ways to use important materials or resources more efficiently and sustainably and plan the transition to more sustainable systems of production and use (Mudd, 2021; UNE, 2020).

Material flow analysis (MFA) is a broad terminology encompassing any quantitative method used to map and quantify flows and stocks of a material of interest in a well-defined system. MFA is applicable to all materials and a range of economic, environmental, and social scales, from a company's supply chain to entire countries and economies. MFA studies have been conducted on metals, including steel and aluminum (Bertram et al., 2009; Cullen et al., 2012), non-metals such as glass and concrete (Westbroek et al., 2021; da Costa Reis et al., 2019), for entire countries (Matsubae-Yokoyama et al., 2009), on a global scale (Miatto et al., 2017), and for multiple materials across whole economies (Fischer-Kowalski et al., 2011). Common to all MFA models is the precise definition of a system, which includes the system boundary, the processes within this system, as well as stock and flow variables representing the storage within and movement between the processes. A physical assumption common to all MFA models is the conservation of mass, where the outflows and inflows of a process must balance with its change of stock.

Typically, in static MFA problems, it is difficult or impossible to collect data for all the flow and change in stock variables inside the system. This gives rise to an under-determined system, in the sense that the size of available data combined with physical constraints, containing at least a mass balance of processes, is still less than the number of model parameters. This places MFA in the domain of high dimensional statistics (Lederer, 2022), which is statistically challenging as the number of unknown parameters exceeds the sample size (Wainwright, 2019). Well-known statistical methods in this area include ridge regression, the LASSO (least absolute shrinkage and selection operator), elastic net, as well as Bayesian approaches (Hoerl & Kennard, 2000; Kruschke, 2015; Tibshirani, 1996; Zou & Hastie, 2005).

Bayesian statistics is particularly well suited for MFA for three main reasons. First, Bayesian statistics works naturally in the under-determined setting by providing a posterior probability over all possible solutions instead of a single solution. Second, while commonly there is a lack of data in MFA problems, there is often domain knowledge or expert opinion that can be used to greatly improve the prediction accuracy of the model, and the Bayesian prior distribution provides a natural framework to incorporate such domain knowledge, which is missed in the traditional MFA approach. Third, Bayesian statistics rigorously quantifies uncertainty in the data and propagates it into the posterior distribution. Additionally, authors including Brunner and Rechberger (2004) and Lupton and Allwood (2018) argued for an incremental approach to MFA, where the system diagram and data are continuously refined and improved until the required level of data certainty has been achieved. The Bayesian paradigm naturally facilitates this through iterative learning of models as new data become available by rerunning the model with new data since the posterior distribution from a previous analysis can be interpreted as the prior distribution for the subsequent analysis.

Non-Bayesian approaches to uncertainty quantification in MFA include works such as Laner et al. (2015), Schwab et al. (2016), and Schwab and Rechberger (2017). These approaches focus on qualitative and quantitative methods of assigning confidence to data or mass-balanced flows but do not provide a modeling procedure that combines prior belief with data and mass balance to produce flow estimates, nor a framework for propagating uncertainty. Bayesian inference provides a modeling procedure that is able to propagate uncertainty and a rigorous, probabilistic interpretation of uncertainty. The popular software “STAN” (Cencic, 2016) uses least squares minimization to conduct data reconciliation of MFA systems. However, STAN requires nonlinear data to be approximated by first-order Taylor expansion, and a normality assumption is made on flows, which can permit negative flow values that are not physically meaningful.

### Previous work on Bayesian approaches to MFA

The use of Bayesian statistics in MFA is still relatively limited. Perhaps the earliest use of Bayesian statistics in MFA is by Gottschalk et al. (2010), where the authors proposed a model where the mass balance equations are parametrized using specific flow ratios (known as transfer coefficients) in the system and tested the model on a case study of flows of concentrations of nanoparticles in Switzerland using simulated data. In Lupton and Allwood (2018), the authors used the same mass balance parametrization as Gottschalk et al. (2010), but with Dirichlet priors on the transfer coefficients rather than uniform or triangular priors, using a Hamiltonian Monte Carlo (HMC)-based sampler called the No-U-Turn Sampler (NUTS) algorithm to sample from the posterior and conducted a case study mapping global steel flows. Recently, Dong et al. (2023) proposed a method that combines prior information from multiple experts in the MFA setting, demonstrating it using the mass balance parametrization of Gottschalk et al. (2010) and Lupton and Allwood (2018). However, when working with the model of Lupton and Allwood (2018) in practice, we found that many divergent samples were produced with the NUTS algorithm, suggesting the posterior samples have not converged. Dong et al. (2023) also encountered divergent samples in their study when using HMC, and opted to use sequential Monte Carlo (SMC) instead to attempt to circumvent this problem. However, it is unclear that the samples from SMC are better converged than the samples from HMC since SMC does not have access to the same divergent sample diagnostics as HMC. Furthermore, SMC appears to significantly increase computational time.

Cencic and Frühwirth (2015) developed a linear Bayesian data conciliation method that can be applied to MFA systems. A subsequent paper by Cencic and Frühwirth (2018) extended this method to include nonlinear constraints. These methods instead parametrize the model directly in terms of the flow variables, by partitioning the set of flow variables in the system into “free variables” $\bf {v_f}$ and “dependent variables” $\bf {v_d}$, where the mass balance equations can be expressed as a relationship between the free variables and the dependent variables. For example, in the linear case, $\bf {v_d}=-D\bf {v_f}-\bf {d}$ can be obtained via Gaussian elimination for some constant matrix $D$ and vector $\bf {d}$. In both papers, the methodology was tested on low-dimensional, simulated examples, with Metropolis Hastings (MH) used as the sampling algorithm on the free variables, with the prior distribution $f(\bf {v_f)})$ of the free variables as the proposal distribution. However, even in low-dimensional examples, it was reported in Cencic and Frühwirth (2015) that the MH sampler can have a very low acceptance probability. Therefore, it is unclear whether the method works well in high-dimensional, under-determined systems with hundreds of flow and change in stock variables, which is important since these systems are typical in MFA. In high dimensions, it becomes increasingly unlikely for proposals from the MH sampling algorithm to satisfy both the mass balance conditions and non-negativity of flow variables. To see this, $\bf {v_d}=-D\bf {v_f}-\bf {d}$ does not guarantee every component of $\bf {v_d}$ will be positive for arbitrary fixed $D$ and $\bf {d}$. As the dimension of $\bf {v_d}$ increases, it becomes increasingly likely that randomly sampled proposal $\bf {v_f}$ during MH will lead to at least one component of $\bf {v_d}$ to be negative, causing the proposal to be in a region of zero posterior probability and the Markov chain Monte Carlo (MCMC) algorithm to be stuck at the current value. More generally, sampling from constrained posteriors is known to be challenging (Lan & Kang, 2023), and HMC also has difficulties when encountering regions of zero posterior probability formed by the constraints (Hoffman & Gelman, 2014).

### Scope of paper

This paper continues the development of Bayesian methodology for MFA. To address the aforementioned computational issues, we relax the mass balance conditions via a noise term. This has the effect of the (approximate) mass balance conditions no longer requiring zero posterior probability in the parameter space where the exact mass balance is not satisfied, making the posterior easier to sample using MCMC algorithms. We show computationally with an aluminum cycle case study that this leads the NUTS sampling algorithm to converge well in high dimensions. Simultaneously, the noise term can be interpreted as a way of modeling epistemic uncertainty in the system, which is likely to be present as MFA systems are simplified rather than perfect representations of reality (Schwab et al., 2016, 2016). It is similar to the concept of “phantom flows,” which is used to account for unexplainable mass imbalances in MFA studies (e.g., Reck et al., 2008). The variance of the noise term can be chosen to be small, so good approximate mass balance is still achieved when there is high confidence in the system definition.

We introduce a child and parent process parametrization framework to model systems with multiple layers of disaggregation in processes and flows, which is a common feature in material flow datasets (Myers et al., 2019, 2019) but has not been considered in previous Bayesian MFA studies. To this end, we assign priors directly on flow mass and change in stock variables in the material system while retaining the ability to incorporate ratio data between arbitrary flows. We demonstrate our method on a high-dimensional aluminum material flow system where a change in stock and disaggregation of processes and flows are simultaneously present.

We illustrate how Bayesian posterior predictive checks can reveal inconsistencies in the data and aid hyperparameter selection. We also show that posterior distribution can inform data collection strategies by identifying which flow or changes in stock variables in the system retain the most uncertainty.

Previous works have not investigated under what conditions estimates and uncertainty quantification produced by Bayesian MFA are reliable. This is important as strong theoretical guarantees that hold in low-dimensional parametric models, such as the Bernstein–von Mises theorem (Vaart, 1998), can fail to hold in high-dimensional settings (Johnstone, 2010) such as the present MFA setting. We address this gap by examining how Bayesian MFA performs on a high-dimensional aluminum case study under relatively weak assumptions, namely weakly informative prior and significant data gaps. In the supporting information, we also conducted a simulation study on a zinc cycle to examine the estimation accuracy and uncertainty quantification of the posterior distributions of our model from a frequentist perspective, which treats probabilities as long-term frequencies.

## METHODS

In this section, we present the details of our proposed Bayesian MFA methodology. Our model produces estimates and uncertainty quantification of all flow and change in stock variables of interest in any given material flow system, in the form of a posterior distribution, which mathematically combines prior domain knowledge and expert opinion with available data while simultaneously propagating uncertainty.

An MFA analysis begins with the definition of a *system diagram*, a graph-like structure of nodes representing $processes$, where the material of interest can be stored as stock, and edges representing $flows$ of the material of interest between processes. Notably, however, flows in both directions between any two processes are permitted. The system diagram will also contain a system boundary, which is used to describe the flows between the system and some external environment. This framework is quite broad and allows processes within a system to not only represent a physical manufacturing process (such as components of a blast furnace) but also examples such as the Earth's lithosphere, the environment, or various usage outlets like households where the material of interest can be stored or flow in and out of. Similarly, the system scope can range from a small supply chain to a global flow of a metal such as zinc. The level of detail of the system diagram is chosen by the modeler to fit the scope of the problem being examined.

Often in MFA problems, certain processes in the system can be disaggregated into constituent subprocesses (see, e.g., Myers et al., 2019). We define a $$parent$$$$process$$ to be a process that contains subprocesses, and a $$child$$$$process$$ be a process which contains no subprocesses instead. By definition, parent and child processes form a partition of the set of all processes in the system. The MFA practitioner should decide the level of disaggregation of each parent process according to their requirements, but parent processes where data are only available on some of its child processes may still be worth disaggregating to incorporate additional data into the model.

The parent and child process structure is useful for modeling the disaggregation of processes. To see this, we assume the stocks and flows of any parent process can be expressed as a linear combination of its constituent child processes. Under this parent and child process framework, multiple levels of disaggregation of processes can be reduced to just two levels (the set of parent processes and the set of child processes), which greatly simplifies modeling of the MFA system. A simple example illustrating the parent and child process framework can be found in the supporting information.

### Formulation of the physical model

Suppose there are $m$ child processes in the system, indexed by $P_0, P_1, \dots P_{m-1}$. Let $s_i(t)$ be the stock variable associated with the process $P_i$, denoting the amount of stock in process $P_i$ at time $t$. In practice, material flow data are typically recorded as the total amount of flow over a period of time (such as on a monthly or yearly basis), so let $U_{j,i}$ represent the total amount of flow of the material of interest from process $j$ to process $i$ during the time period $t-\Delta t$ to $t$. Here, $\Delta t$ represents the period during which the total amount of flow was reported, which can, for example, be in months or years. Note that typically in MFA systems, not all processes necessarily possess a stock variable, for example, if the physical process which it is modeling does not contain a physical stock of the material of interest. Similarly, most processes will not receive flows from or flow to every other process, so the notations introduced in this paragraph, such as $s_i$ and $U_{j,k}$, are understood to be over existing stocks and flow variables only. For each process $P_i$, we assume mass is conserved between its stock, inflows, and outflows over the time period $t-\Delta t$ to $t$, which we formulate as:

1 $$ S_i=s_i(t)-s_i(t-\Delta t) = \sum _{j} U_{j,i}-\sum _{k} U_{i,k}.$$

The left-hand side $S_i=s_i(t)-s_i(t-\Delta t)$ is simply the change in stock during the time period $t$ to $t-\Delta t$. In this paper, we only consider stationary models at a snapshot of time $t$, so the variables of interest in the model are $S_i$ and $U_{j,k}$. For more compact notation, let $\bf {S}$ be the vector of $q$ change in stock variables $S_i$ of the child processes in the system, and $\bf {U}$ a vector of $p-q$ flow variables $U_{j,k}$ of the child processes in the system (for a total of $p$ variables). Note, because flows and changes in stock of parent processes can be expressed linearly in terms of its constituent child processes, conservation of mass for all child processes automatically implies conservation of mass for all parent processes. Similarly, Bayesian modeling only needs to be conducted on the child processes, as the posterior samples on flow and stocks change variables of parent processes can be obtained by summing the posterior samples of the constituent child processes.

### Data structure

Data in MFA can in principle be any arbitrary function $f(\bf {S},\bf {U})$ of the variables of interest $\bf {S}$ and $\bf {U}$. However, data and mass balance conditions typically come in the following four forms, which we can express in terms of $\bf {S}$ and $\bf {U}$:

1. Observations of changes in stock of child processes or observations of flows between child processes. For example, in Figure 2, $S_0=-37.2$ Mt could be used to describe the change in stock of the “Reserves” process, while the flow from “Reserves” to “Mining” can be described by $U_{0,1}=37.2$ Mt. Here, the process “Reserves” is labeled by $P_0$ and the process “Mining” by $P_1$.
2. Observations of changes in stock of parent processes, or observations of flows between processes where at least one process is a parent, which can be treated as the sum of multiple flows. For example, the combined flow of 9.8 Mt in Figure 2 from “WasteManagement” to “Recycling” consists of “WasteManagement” as the origin process, and the destination processes are “Remelting” and “Refining,” two of the subprocesses of “Recycling.” This flow can be represented by the sum of flows $U_{31,7}+U_{31,8}=9.8$ Mt. Here, we used $P_{31}$ to denote “WasteManagement,” and $P_{7}, P_{8}$ “Remelting” and “Refining” subprocesses of “Recycling,” respectively. So, for example, $U_{31,7}$ represents the flow from “WasteManagement” to “Remelting.”
3. Conservation of mass. For child process $i$, this is represented by Equation (1).
4. Ratio data between flows or sums of flows, for instance, transfer coefficients:

2 $$ \frac{U_{i,j}}{{\sum _{k} U_{i,k}}}=\alpha _{i,j},$$

where $\alpha _{i,j}$ is a known transfer coefficient of the flow from process $i$ to process $j$ and is defined as the ratio between the flow from process $i$ to process $j$, divided by the total outflow of process $i$. In case studies of this paper, we only consider transfer coefficients of processes without a change in stock variable. In general, a change in stock can be split into two flows (flow into and out of stock), which allows transfer coefficients to be calculated. We also consider ratios between aggregated flows as an extension beyond standard transfer coefficients.

Ratio data can also be alternatively parametrized linearly in the following way:

3 $$ U_{i,j}-\alpha _{i,j}{\sum _{k} U_{i,k}}=0.$$

We note it is possible to conveniently formulate all the most common forms of data and mass balance conditions in MFA in terms of linear relationships between the change in stock variables $S_i$ and the flow variables $U_{j,k}$, even when the system contains disaggregation of processes. However, for a more general framework and to demonstrate our model can incorporate nonlinear data as well, we choose to parametrize ratio data as Equation (2) when evaluating the model on the aluminum case study in Section 3.

Using more compact notation, the relationship between the flow and change in stock variables and the data and physical constraints can be represented by the following model:

4 $$\bf {Y}=\def\eqcellsep{\;}\begin{bmatrix} X\bf {\theta } \\ \bf {R}(\bf {\theta }) \end{bmatrix} +\bf {\epsilon }, \quad \bf {\theta }=\def\eqcellsep{\;}\begin{bmatrix} \bf {S} \\ \bf {U} \end{bmatrix}$$

where $\bf {\theta }$ is a concatenated vector of $\bf {S}$ and $\bf {U}$ of length $p$, representing all the child flow and change in stock variables of the system. $\bf {Y}$ is a vector of length $n$ of observed values or 0 for mass balance conditions. $X$ is a design matrix representing linear data and mass balance conditions, and $\bf {R}(\bf {\theta })$ a vector representing nonlinear data. $\bf {\epsilon }$ is a random vector (of length $n$) representing uncertainty in the data, which could be caused by measurement or rounding errors. Note, in the MFA setting, it is often the case that $n \ll p$.

The goal of any Bayesian model is to obtain posterior distributions over the variables of interest, in this case $\bf {\theta }$, to perform inference such as point estimation or uncertainty quantification. In the following section, we describe how to construct the prior for our model, the form of the likelihood, and how to obtain the posterior of $\bf {\theta }$ via Bayes's theorem.

### Bayesian model detail

Bayesian inference is a statistical framework in which Bayes's theorem, a fundamental result in probability theory, is used to update one's beliefs regarding parameters of interest $\bf {\theta }$ based on new data or evidence $\bf {Y}$. Mathematically, the prior distribution $p(\bf {\theta })$ is used to express one's belief prior to seeing the data and the likelihood function $p(\bf {Y}|\bf {\theta })$ used to express the probability of observing the data. The goal in Bayesian inference is to obtain the posterior distribution $p(\bf {\theta }|\bf {Y})$, which represents the state of one's updated beliefs after observing the data. This is done via Bayes's theorem:

5 $$ p(\bf {\theta }|\bf {Y})=\frac{p(\bf {\theta })p(\bf {Y}|\bf {\theta })}{\int p(\bf {\theta })p(\bf {Y}|\bf {\theta }) d \bf {\theta } }.$$

Most posterior distributions do not have a closed form expression due to it not being possible to evaluate $\int p(\bf {\theta })p(\bf {Y}|\bf {\theta }) d \bf {\theta }$ in closed form. MCMC methods are computational algorithms used to sample from distributions that do not have a closed form, including posterior distributions. MCMC methods construct a Markov chain that converges to the target distribution by iteratively sampling from a proposal distribution and only accepting samples that satisfy suitable criteria that suggest they could be feasibly generated from the target distribution.

**FIGURE 1:**
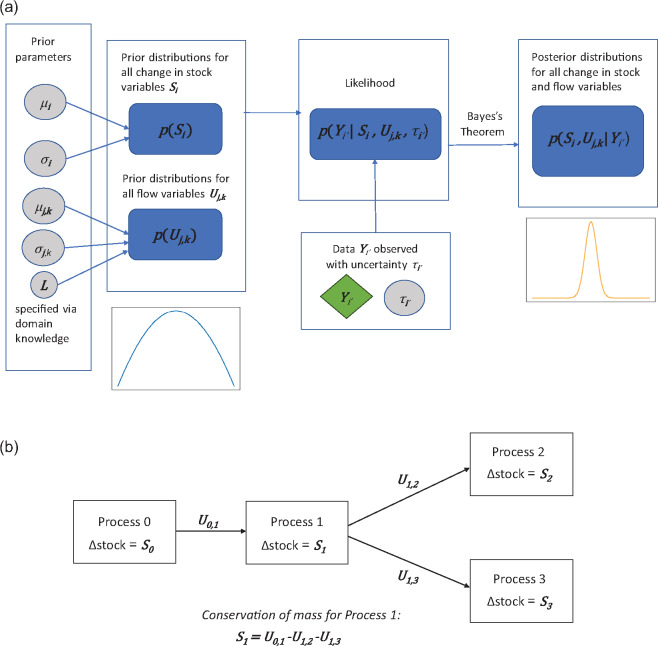
Schematic of the Bayesian model (a), example of simple material flow analysis (MFA) system (b). The circles colored in gray represent noise parameters or prior hyperparameters, the diamond colored in green represent the data, and the rounded rectangles colored in blue represent distributions over the variables of interest as well as the model likelihood. Definitions for each variable and parameter in this figure can be found in Section 2.

For our Bayesian model (see Figure 1 for model schematic), we consider normal priors for the change in stock variables $S_i$, which reflects the fact that the change in stock can be both positive and negative. For the flow variables $U_{j,k}$, we consider truncated normal priors for the flow variables on the positive interval $[0,L]$, where $L$ is chosen based on domain knowledge of the scale of flows inside the system being modeled, or simply chosen to be positive infinite if none is available. This is to ensure the posterior distribution of the flow variables is positive for nonnegative values only as flow quantities cannot be negative in reality. Furthermore, the normal and truncated normal distributions are flexible, in the sense that an informative prior distribution can be assigned by choosing the prior mode as a confident estimate (from expert knowledge) and choosing a small prior variance to create a narrow distribution around the prior mode. On the other hand, an uninformative prior distribution can be assigned by choosing a large prior variance around a rough guess for the prior mode instead.

6 $$S_i \sim \mathcal {N}(\mu _i, \sigma _i^2),\quad U_{j,k} \sim \mathcal {TN}(\mu _{j,k}, \sigma _{j,k}^2, 0, L),$$

where $\mu _i$ and $\sigma _i^2$ are the prior mean and variance of $i$th change in stock variable $S_i$, respectively, and $\mu _{j,k}$ and $\sigma _{j,k}^2$ the corresponding prior hyperparameters, respectively, for the truncated normal distribution for the flow variable $U_{j,k}$, representing the flow quantity from process $j$ to process $k$. We also assume prior variables are independent, so the overall prior distribution $p(\bf {\theta })$ over the change in stock and flow variables is of the form:

7 $$p(\bf {\theta }) \propto \prod _{i} \frac{1}{\sigma _i}\exp \left(\frac{-(S_i-\mu _i)^2}{2\sigma _i^2}\right) \prod _{(j,k)} \frac{1}{\sigma _{j,k}}\exp \left(\frac{-(U_{j,k}-\mu _{j,k})^2}{2\sigma _{j,k}^2}\right) \mathrm{I}_{U_{j,k} \in [0,L]},$$

where $\mathrm{I}_{U_{j,k} \in [0,L]}$ is an indicator function, and the products are over the change in stock variables $S_i$ and flow variables $U_{j,k}$.

For the likelihood, we again use a truncated normal likelihood for any data on observed flow values to ensure the data generated by the likelihood is plausible (since flow data should be strictly positive) and a normal likelihood for data on observed change in stock values and general nonlinear data. We include an additional normally distributed noise term in the mass balance conditions mainly for practical reasons: the noise term reduces the region of zero posterior probability, which makes sampling from the posterior computationally much more tractable. Additionally, the noise term has the interpretation of modeling epistemic or systematic uncertainty in the MFA system. In theory, every process should be exactly mass-conserved if the system definition and diagram are a perfect representation of the underlying real system being modeled. However, in reality, the system diagram and definition are simplified and approximate models of reality, and so it is reasonable to account for uncertainty in the underlying system itself, which can be modeled through a noise term in the mass balance conditions. In practice, flows reported in MFA studies are rarely exactly mass-balanced, so likewise a Bayesian MFA approach does not need to produce perfectly mass-balanced flows to provide useful estimates.

8 $$Y_{i^{\prime }}|\bf {\theta } \; \sim \mathcal {N}(\bf {x_{i^{\prime }}}^\top \bf {\theta }, \tau _{i^{\prime }}^2) \quad \rm{for observations} \ Y_{i^{\prime }} \ \rm{on change in stock data}$$

9 $$Y_{j^{\prime }}|\bf {\theta } \; \sim \mathcal {TN}(\bf {x_{j^{\prime }}}^\top \bf {\theta }, \tau _{j^{\prime }}^2, 0, \infty) \quad \rm{for observations} \ Y_{j^{\prime }} \ \rm{on flow data}$$

10 $$Y_{k^{\prime }}|\bf {\theta } \; \sim \mathcal {N}(R(\bf {\theta })_{k^{\prime }}, \tau _{k^{\prime }}^2) \quad \rm{for observations} \ Y_{k^{\prime }} \ \rm{on nonlinear data}$$

11 $$Y_{l^{\prime }}|\bf {\theta } \; \sim \mathcal {N}(\bf {x_{l^{\prime }}}^\top \bf {\theta }, \tau _{l^{\prime }}^2) \quad \rm{for} \ Y_{l^{\prime }}=0 \ \rm{on mass balance conditions}$$

Here, $Y_{i^{\prime }}$ is the $i^{\prime }$th row or entry of the observation vector $\bf {Y}$ and $\bf {x_{i^{\prime }}}^\top$ the $i^{\prime }$th row of the design matrix $X$, $R(\bf {\theta })_{k^{\prime }}$ the $k^{\prime }$th row of the vector $\bf {R}(\bf {\theta })$, and $\tau _{i^{\prime }}^2$ representing variance of the noise of the $i^{\prime }$th observation, which are assumed to be independent. The overall likelihood $p(\bf {Y}|\bf {\theta })$ is therefore of the form:

12 $$p(\bf {Y}|\bf {\theta })=\prod _{i^{\prime }} p(Y_{i^{\prime }}|\bf {\theta })\prod _{j^{\prime }} p(Y_{j^{\prime }}|\bf {\theta })\prod _{k^{\prime }} p(Y_{k^{\prime }}|\bf {\theta })\prod _{l^{\prime }} p(Y_{l^{\prime }}|\bf {\theta }),$$

where

13 $$p(Y_{i^{\prime }}|\bf {\theta }) \; \propto \frac{1}{\tau _{i^{\prime }}}\exp \left(\frac{-(Y_{i^{\prime }}-\bf {x_{i^{\prime }}}^\top \bf {\theta })^2}{2\tau _{i^{\prime }}^2}\right),$$

14 $$p(Y_{j^{\prime }}|\bf {\theta }) \; \propto \frac{1}{\tau _{j^{\prime }}}\exp \left(\frac{-(Y_{j^{\prime }}-\bf {x_{j^{\prime }}}^\top \bf {\theta })^2}{2\tau _{j^{\prime }}^2}\right) \mathrm{I}_{Y_{j^{\prime }} \in [0,\infty)},$$

15 $$p(Y_{k^{\prime }}|\bf {\theta }) \; \propto \frac{1}{\tau _{k^{\prime }}}\exp \left(\frac{-(Y_{k^{\prime }}-R(\bf {\theta })_{k^{\prime }})^2}{2\tau _{k^{\prime }}^2}\right),$$

16 $$p(Y_{l^{\prime }}|\bf {\theta }) \; \propto \frac{1}{\tau _{l^{\prime }}}\exp \left(\frac{-(Y_{l^{\prime }}-\bf {x_{l^{\prime }}}^\top \bf {\theta })^2}{2\tau _{l^{\prime }}^2}\right).$$

Given the prior and likelihood, we can use Bayes's Theorem 5 to obtain the posterior distribution $p(\bf {\theta }|\bf {Y})$. However, the posterior induced by this model does not admit an analytical form, and so we employ the No-U-Turn Sampler (NUTS) algorithm of Hoffman and Gelman (2014) to sample from the posterior, implemented via the PyMC3 library (Salvatier et al., 2016) in Python. NUTS is an HMC that achieves increased sampling efficiency over traditional MCMC methods, such as MH, by exploiting the gradient information of the target distribution to generate more informed sample proposals and explore the target distribution more efficiently. This is especially important in high dimensions (which applies to many MFA systems) since the probability mass of the target distribution is more likely concentrated in smaller regions, which is inefficient to explore via MH due to random walk behavior.

### Posterior predictive checks

For Bayesian modeling of MFA systems, we recommend performing posterior predictive checks to verify whether data generated by the model are similar to the observed data and adequately mass conserved, which gives some assurance that the prior, model, and parameters chosen are sensible. Here, we briefly describe the method of posterior predictive checking outlined in Chapter 6 of Gelman et al. (2013). Recall that, in our model, the observation vector $\bf {Y}$ represents the observed data (such as on flows or change in stocks), as well as physical conditions such as mass balance and $\bf {\theta }$ the vector of parameters. Let $\bf {Y^{rep}}$ be replicated data that could have been observed; the distribution of $\bf {Y^{rep}}$ given the observed data $\bf {Y}$, also known as the posterior predictive distribution (PPD), is given by:

17 $$ p(\bf {Y^{rep}} | \bf {Y})=\int p(\bf {Y^{rep}} | \bf {\theta })p(\bf {\theta } | \bf {Y}) \,d \bf {\theta }.$$

Typically, the check is done on suitable scalar test quantities $T(\bf {Y}, \bf {\theta })$, chosen based on the real problem being modeled. The test quantity of the replicated data $T(\bf {Y^{rep}}, \bf {\theta })$ is compared with the test quantity of the observed data $T(\bf {Y}, \bf {\theta })$ through statistical tests or graphical checks to look for systematic discrepancies between the simulated and originally observed data. For our Bayesian MFA model, we choose the test quantities to be each individual observed data and mass balance conditions of child processes in the system; in other words, we choose test quantities $T_i(\bf {Y}, \bf {\theta })=Y_i$ for each $i$. This ensures we minimally check using the posterior predictive distribution that the model is consistent with the existing data as well as the mass balance of child processes. One way to compare the observed data with the posterior predictive distribution is to calculate the Bayesian posterior predictive *p*-values (${\it pval}_i$) for the test statistic, in our case the marginal observations $Y_i$, which are given by

18 $$ pval_i=P(Y_i^{rep} \ge Y_i | \bf {Y})=\int \int \mathrm{I}_{Y_i^{rep} \ge Y_i} p(\bf {Y^{rep}} | \bf {\theta })p(\bf {\theta } | \bf {Y}) \,d\bf {Y^{rep}} d \bf {\theta }.$$

A very large or small *p*-value (e.g., greater than 0.95 or smaller than 0.05 as suggested by Gelman et al. (2013)) suggests the observed test quantity is unlikely to be replicated in repeated experiments if the model was true, implying there is an inconsistency between the model and the data. We also calculate the posterior predictive marginal $95\%$ highest density interval for each $Y_i | \bf {Y}$ to see if they contain the observed values $Y_i$ (which include the conservation of mass conditions where $Y_i=0$).

In practice, the integrals in Equations (17) and (18) are analytically intractable, so we again approximate the posterior predictive distribution via sampling. Specifically, we simulate one sample of $\bf {Y^{rep}}$ from the posterior predictive distribution for each posterior sample of $\bf {\theta }$, and we approximate *p*-values of Equation (18) by checking the proportion of the posterior predictive samples of $Y_i^{rep}$ that exceed the observed value $Y_i$, for each $i$.

### Hyperparameter and noise parameter selection

In general, the prior hyperparameters $\mu _i$, $\mu _{j,k}$ should be specified through domain knowledge to reflect the modeler's best estimate of the stock change and flow variables a priori, and $\sigma _i^2$, $\sigma _{j,k}^2$ chosen to reflect prior uncertainty; the less confident the estimates for $\mu _i$, $\mu _{j,k}$, the larger $\sigma _i^2$, $\sigma _{j,k}^2$.

The noise variance parameters $\tau _{i^{\prime }}^2$ should also ideally be chosen to reflect uncertainty in the data, and in the case of the mass balance conditions, epistemic uncertainty in the system definition. We recommend using posterior predictive checking to help select suitable noise parameter values, especially if no knowledge of the degree of data uncertainty is available. One can start with a small choice of standard deviation parameters (such as $10\%$ of the observed data value and a small constant for the mass balance conditions), run the model and conduct posterior predictive checks, and identify the data points and mass balance conditions that exhibit extreme Bayesian *p*-values. The standard deviation parameter for those data points or mass balance conditions should be increased and the model rerun until no extreme Bayesian *p*-values remain.

Full details of hyperparameters choice in the case studies examined in Section 3 can be found in the supporting information. We give examples of how to specify prior hyperparameters in the case where there is weakly informative domain knowledge where some flows are known to the nearest order of magnitude, as well as flows where there is no prior knowledge available.

## RESULTS

In this section, we demonstrate our Bayesian MFA method on an aluminum cycle containing significant disaggregations of processes and flows. We evaluate our model on the aluminum cycle under a weakly informative prior for two different levels of data availability and compare the results. We also present posterior predictive checks to identify inconsistencies between the model and data and processes that are not mass-balanced by the available data.

### Aluminum cycle

**FIGURE 2:**
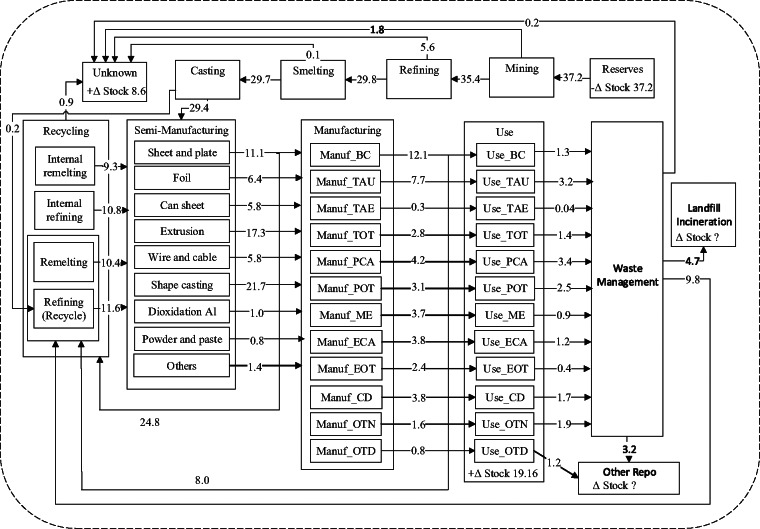
Global anthropogenic metallurgical aluminum cycle in 2009. *Source*: Adapted from fig. 1 of Liu et al. (2013). The mass of aluminum is measured in megatonnes (Mt). BC, Building & Construction; TAU, Transportation-Auto & Lt Truck; TAE, Transportation-Aerospace; TOT, Trans-Other; PCA, Packaging-Cans; POT, Packaging-Other; ME, Machinery & Equipment; ECA, Electrical-Cable;, EOT, Electrical-Other; CD, Consumer Durables; OTN, Other (ex Destructive Uses); OTD, Destructive Uses.

We evaluate our model on the global anthropogenic metallurgical aluminum cycle in 2009 from Liu et al. (2013). The associated system diagram Figure 2 is adapted from fig. 1 of Liu et al. (2013). The aluminum cycle contains significant disaggregations of processes. For example, the “Use” process contains subprocesses such as “Use_BC” (building and construction) and “Use_ME” (machinery and equipment) representing different use product categories of aluminum. Furthermore, for aggregated processes such as “Manufacturing” and “Recycling,” Figure 2 only displays data on aggregated flows and change in stocks. For example, the flow 12.1 Mt from “Manufacturing” is a combined flow to “Use_BC” and the subprocesses “Remelting” and “Refining” of “Recycling.” So, it is not clear from Figure 2 alone in what proportion this flow should split among the constituent subprocesses. In tables S5, S6 and S8 of the supplementary information, Liu et al. (2013) provide ratios specifying how certain aggregated flows should split into constituent subflows (known as transfer coefficients), specifically for the aggregate flows from “Semi-manufacturing” to “Manufacturing” and “Recycling,” and from “Manufacturing” to “Use” and “Recycling.” For the purposes of testing our model, we treat the values presented in fig. 1 of Liu et al. (2013) and transfer coefficients in tables S5, S6, S8 of the supplementary information as data. We also henceforth use “reported value” to refer to any flow and change in stock values in fig. 1 of Liu et al. (2013), as well as values of disaggregated flow and change in stock values calculated through the transfer coefficients in the supplementary material of Liu et al. (2013).

We evaluate the model under two different scenarios. In scenario A, we deliberately withhold the transfer coefficients in the supplementary information of Liu et al. (2013) from the model and only use the data displayed in Figure 2. Instead, where transfer coefficients are available to calculate the value of the disaggregated flow, we set the prior mode of the disaggregated flow variable to the nearest power of 10 of the reported value; otherwise, an uninformative prior with mode 1.0 Mt is used. The purpose of scenario A is to see if our Bayesian MFA model can still produce useful estimates of flows and changes in stock under a weakly informative prior with a significant amount of missing data. Scenario A also mimics a situation that could be applicable to many MFA problems, where data are available on an aggregated/parent level but not the disaggregated/child level, and a rough estimate like the order of magnitude of the flows and changes in stock on the disaggregated level is obtained from surveying domain experts or approximate calculations, which can be used to construct a weakly informative prior. In scenario B, the same weakly informative prior is used, but the flow ratios in the supplementary information of Liu et al. (2013) are provided to the model as well. Scenario B mimics a situation toward the end of an MFA analysis, when data are available for most of the flow and change in stock variables in the system. In both scenarios, we assume a low degree of epistemic uncertainty in the system diagram and set the standard deviation of mass balance conditions to a constant 0.05 Mt. This choice leads to well-mass-balanced posterior means and samples of the flow and change in stock variables, and we provide an analysis of posterior mass balance conditions in the supporting information (section S5) for full detail. Full detail of prior hyperparameters can also be found in the supporting information (section S4).

**FIGURE 3:**
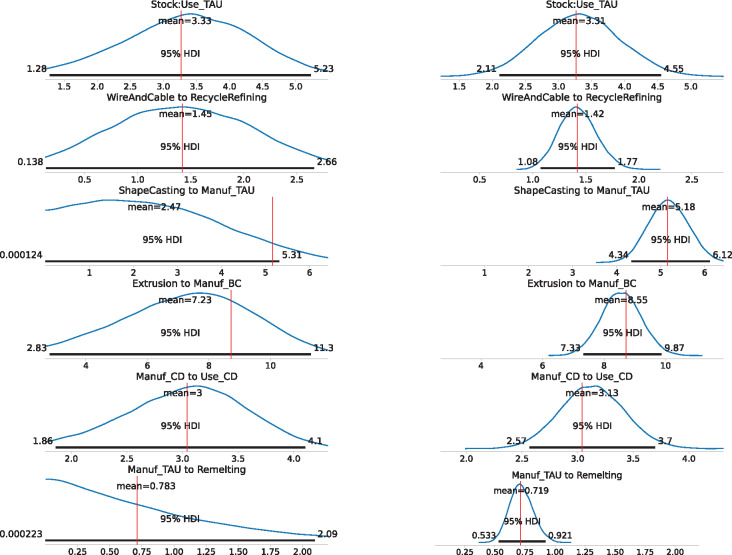
Marginal posterior distributions for a selection of flow and change in stock variables of the aluminum dataset. For each flow or change in stock variables, we display both the marginal posterior for scenario A (on the left) and for the scenario B (on the right). Each marginal posterior plot displays the mean and the 95% highest density interval (HDI). The red vertical line in each graph represents the reported value of the variable, calculated using transfer coefficients provided in the supplement of Liu et al. (2013). Refer to Figure 2 for process name definitions. Underlying data for this figure can be found at https://github.com/jwang727/BayesianMFA.

Figure 3 displays a selection of marginal posterior distributions for flow and change in stock variables of disaggregated/child processes in the aluminum cycle modeled here. Only a selection is displayed here for brevity as there are around 180 flows or change in stock variables in the model. Under scenario A, the marginal posteriors are relatively more biased away from the reported value, which is not unexpected as data on the disaggregated level were withheld from the model in scenario A. Nevertheless, all of the reported values of flows and change in stock (when they are available in the supplement of Liu et al. (2013)) are contained within the $95\%$ posterior marginal highest density intervals (HDIs). A small number of reported values are near the edge of the HDI like the flow “ShapeCasting to Manuf_TAU.” This could be caused by the reported value (around 5.1 Mt) being close to the middle of the nearest orders of magnitudes (1.0 and 10.0 Mt), which is more difficult for a prior based on the nearest order of magnitude (1.0 Mt in this case) to capture without actual data on the flow. Overall under scenario A, the posterior estimates and uncertainty quantification for disaggregated flows or change in stock variables obtained still capture the reported values reasonably well despite not being given data on any disaggregated flows. With the addition of transfer coefficient data under scenario B, the posterior marginal distributions generally possess narrower HDIs compared to scenario A and are centered much more around the reported value, and again the reported values are contained within the $95\%$ posterior HDI. Quantitatively, the average length of the $95\%$ posterior HDI overall flow and change in stock variables under scenario A is 2.15 Mt, while under scenario B is 1.46 Mt.

In both scenarios, it took the NUTS algorithm around 90 min to generate 24,000 posterior samples across two chains on an Intel i5-1145G7 CPU, 2.6GHz. The traceplots and convergence checks suggest the NUTS algorithm has converged, and we did not observe any divergent samples. A selection of traceplots and further diagnostics can be found in section S1 of the supporting information. Posterior pairplots illustrating the posterior correlation between a selection of flow variables can be found in section S3. In section S6, we include a simulation study on a zinc cycle to examine the estimation accuracy and uncertainty quantification of our model from a frequentist perspective. We demonstrate on a zinc cycle that incorporating even a weakly informative prior can significantly reduce the estimation error, and the posterior credible intervals can consistently contain the true value of flows and change in stocks and be interpreted as confidence intervals.

### Posterior predictive checks on aluminum model

**FIGURE 4:**
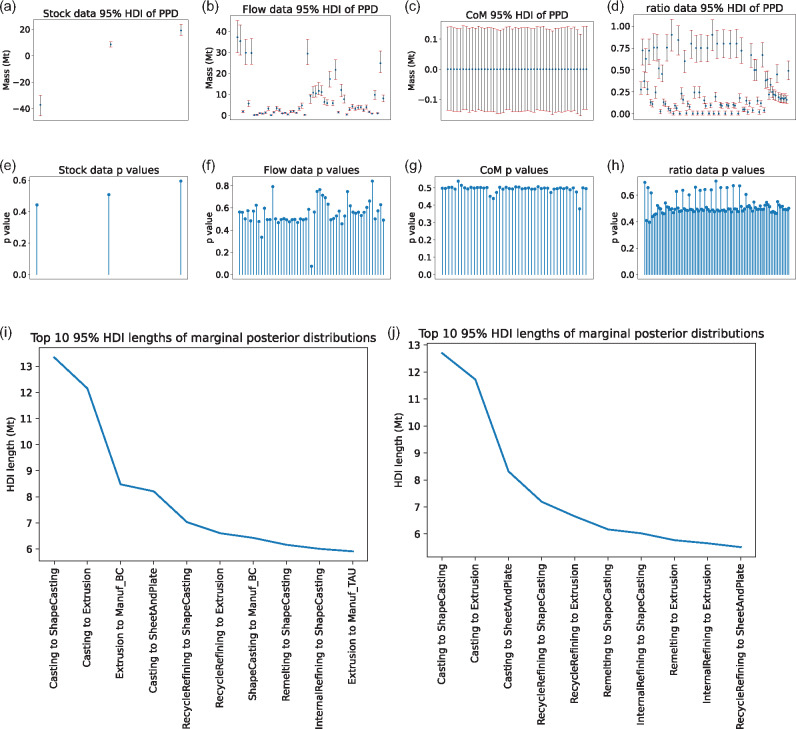
Graphs of posterior predictive checks and posterior predictive highest density interval (HDI) lengths, for scenario B. In the first row, we have the sample $95\%$ posterior predictive HDIs (red bar) and the observed values (blue dot) for the change in stock data, observed flow data, conservation of mass conditions, and flow ratio data, respectively. In the second row, we have the posterior predictive *p*-values for the observed change in stock variables, observed flow variables, conservation of mass conditions, and flow ratio data, respectively. In the third row, we plot the top 10 largest marginal posterior HDI lengths for scenario A (left) and scenario B (right). Underlying data for this figure can be found at https://github.com/jwang727/BayesianMFA.

In this section, we present some additional results for the aluminum model. First, we apply the posterior predictive checks described in Section 2.4 on the aluminum model under scenario B. From Figure 4a–d, it can be seen that the $95\%$ HDIs all contain the observed values for all change in stock data, flow data, conservation of mass conditions, and ratio data. Moreover, from Figure 4e–h, it can be seen that the posterior predictive *p*-values are mostly between 0.3 and 0.7, and no *p*-value is smaller than 0.05 or greater than 0.95, suggesting that no *p*-values are extreme and the model generally fits the data reasonably well. However, there are a few variables close to extreme values. In particular, the 27th flow data *p*-value in Figure 4f is around 0.07, which represents the total outflow from “Internal remelting” to “Semi-manufacturing” of 9.3 Mt. Upon reviewing the data, it was found that the total inflow of “Internal remelting” only summed to 7.1 Mt. The posterior predictive check therefore helpfully highlighted a discrepancy that suggests the data for the process internal remelting have more sizable mass imbalance. Similarly, the 49th flow data *p*-value is relatively high at around 0.8, which are the data representing the outflow from Manuf_OTD of 0.8 Mt. Upon examining the data, it was found that the total inflow of Manuf_OTD is 1.0.

In MFA, data are expensive to collect, so it is useful to prioritize which flow and change in stock variables to collect more data on. This will likely depend on what questions the modeler is most interested in answering regarding the real system being modeled. Without specific questions, however, Bayesian inference provides default strategies for prioritizing which data points to collect, by ranking the variables in terms of descending posterior uncertainty. We plot the top 10 flow and change in stock variables in descending length of their marginal $95\%$ HDI for both scenarios A (Figure 4i) and B (Figure 4j). In scenario B, the most uncertain variables are mostly flows from “Casting” or “Recycling” to “Semi-manufacturing,” which is expected as those are the disaggregated flows where data are not available. For scenario A, the most uncertain variables are more scattered throughout the system, as scenario A has no data on any disaggregated flows.

## DISCUSSION

This paper presented a novel MFA methodology under the Bayesian framework that addresses existing challenges and expands the applicability of Bayesian inference in MFA. By relaxing the mass balance constraints with a noise term, we improved upon the computational scalability and reliability of posterior samples compared to existing methods, while still retaining well-mass-balanced posterior estimates of stock changes and flows. We introduced a child and parent parametrization that can conveniently deal with MFA systems with multiple layers of disaggregation of processes and flows, providing posterior distributions on flows and change in stocks on all levels of disaggregation in the system, including lower levels where data are often unavailable. We showed that even a weakly informative prior, specifically a prior based on the nearest order of magnitude of stock changes and flows, is capable of greatly improving the model's estimation accuracy and quality of its uncertainty quantification, especially during the early stages of the analysis when there is a lack of data, reaffirming the benefit of a Bayesian approach to MFA. We also demonstrated how posterior predictive checks can be used to check if the model is consistent with the data and mass balance conditions, help identify data inconsistencies, and aid in selecting noise parameter values for the data and mass balance conditions.

Like other Bayesian approaches, our method requires prior distributions of all flow and change in stock variables of interest to be manually specified, which is an additional requirement compared to traditional MFA. However, we argue that this should be a standard part of any MFA, where domain knowledge should be continuously collated, and the prior distribution offers a principled, mathematical way of incorporating this knowledge into the model. The priors used in this paper are unimodal and only weakly informative at most, to reduce requirements on the prior so that it can be applied in a wider range of MFA settings. In principle, more or less informative priors can be used based on domain knowledge of the application. In the presence of multiple expert opinions, mixture priors that combine multiple experts similar to the approach of Dong et al. (2023) can be considered in our model framework.

The Bayesian approach inevitably comes with a higher computational cost, as the full posterior distribution of each variable of interest needs to be calculated rather than just a point estimate, and may run into convergence issues (Betancourt, 2017). The NUTS HMC algorithm used for our model took around 90 min to generate 24,000 posterior samples across two chains for the aluminum dataset, which we consider acceptable. We also note that most studies in the MFA literature do not use very large datasets, so we do not anticipate computational cost to be a major issue. However, for much larger material flow datasets, approximate methods such as variational Bayes can be employed to reduce the computational cost, or minimally just estimating the posterior mode directly (and forgoing uncertainty quantification) can potentially yield better point estimates than a non-Bayesian method if informative priors are available.

## Supplementary Information

**Supporting Information S1**: This supporting information contains additional details on the methodology and computational results.

## Data Availability

The aluminum data used in this paper can be found in Liu et al. (2013). The zinc data used in this paper can be found in Graedel et al. (2005). Details on the choice of prior and model parameters are available in the supporting information of this article. Data used to produce figures in this paper can be found at https://github.com/jwang727/BayesianMFA.
