# Supplementary material for: Bayesian material flow analysis for systems with multiple levels of disaggregation and high dimensional data
Source: J Ind Ecol. 2024 Sep 30;28(6):1409–21. doi: 10.1111/jiec.13550 (PMC11667660; doi:10.1111/jiec.13550)
Supplement: Supplementary file 1 — Supporting Information S1: This supporting information contains additional details on the methodology and computational results. [file 44498_2024_2806007_MOESM1_ESM.pdf]

SUPPORTING INFORMATION FOR:

Wang, J., Ray, K., Brito-Parada, P., Plancherel, Y., Bide, Tom., Mankelow, Joseph., Morley, J., Stege-mann, J., Myers, R. (2024.) Bayesian material flow analysis for systems with multiple levels of disaggre-gation and high dimensional data. *Journal of Industrial Ecology.*

Summary:

This supporting information contains additional details on the methodology and computational results.

Contents

S1 Parent and child processes **2**

S2 Model convergence diagnostics **4**

S3 Posterior pairplots **6**

S4 Detail of prior parameters for the aluminium model **7**

S5 Assessment of posterior mass balance for the aluminium model **9**

S6 Zinc cycle simulation study **10**

S7 Detail of parameters for the zinc models **14**

S8 Gaussian model **15**

S9 Discussion of model parametrisation **18**

S1 Parent and child processes

In this section we elaborate on the parent and child process framework used to model disaggregation of processes or flows in MFA systems, and provide a simple illustrative example. Recall that a child process is any process which does not contain subprocesses, and a parent process is any process which does contain subprocesses. This by definition partitions all processes in the system into either child or parent processes, and all parent processes can be broken down into a set of constituent child processes. This partition of parent and child processes is useful because it allows multiple levels of disaggregation to be modelled using just two types of processes with a bottom up approach. In particular, only the child processes need to be modelled, and the parent processes variables are simply the sum of its constituent child processes variables. To illustrate this, consider the (non mass conserved) simple example of Figure S1:

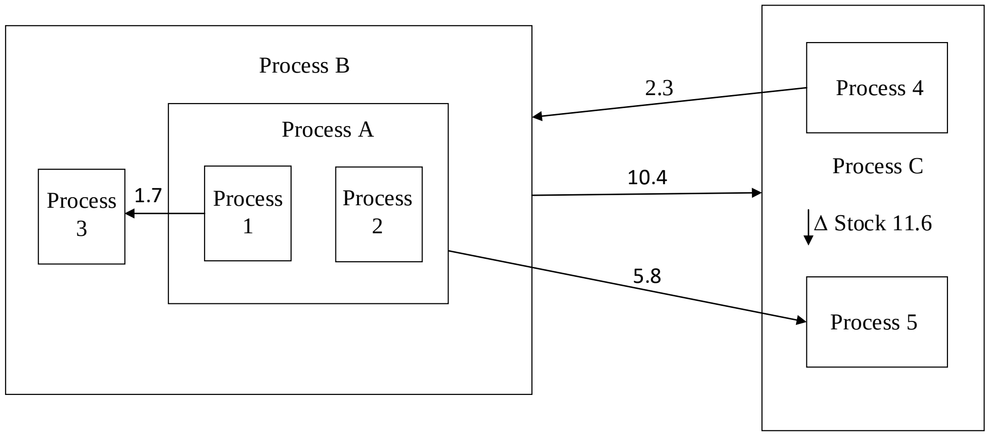

Figure S1: A simple example of a MFA system with aggregation

From a typical top down approach, this example system contains 3 levels of disaggregation, since ‘Process B’ contains ‘Process A’, and ‘Process A’ in turn contains ‘Process 1’ and ‘Process 2’. In our framework however, the child processes are ‘Process 1’,‘Process 2’,‘Process 3’,‘Process 4’ and ‘Process 5’ while the parent processes are ‘Process A’, ‘Process B’ and ‘Process C’. The data in Figure S1 are the flows $U_{1,3} = 1.7$, $U_{4,B} = 2.3$, $U_{B,C} = 10.4$ and $U_{A,5} = 5.8$, and the change of stock $S_C = 11.6$. The change in stock or flow data involving parent or aggregated processes can all be expressed in terms of flows involving child processes:

$$S_C = S_4 + S_5 \tag{S1}$$

$$U_{4,B} = U_{4,1} + U_{4,2} + U_{4,3} \tag{S2}$$

$$U_{B,C} = U_{1,4} + U_{2,4} + U_{3,4} + U_{1,5} + U_{2,5} + U_{3,5} \tag{S3}$$

$$U_{A,5} = U_{1,5} + U_{2,5} \tag{S4}$$

The data in this example can therefore be formulated with the following design matrix:

$$\begin{pmatrix} 0 & 0 & 1 & 0 & 0 & 0 & 0 & 0 & 0 & 0 & 0 & 0 \\ 1 & 1 & 0 & 0 & 0 & 0 & 0 & 0 & 0 & 0 & 0 & 0 \\ 0 & 0 & 0 & 0 & 0 & 0 & 0 & 0 & 0 & 1 & 1 & 1 \\ 0 & 0 & 0 & 1 & 1 & 1 & 1 & 1 & 1 & 0 & 0 & 0 \\ 0 & 0 & 0 & 0 & 1 & 0 & 1 & 0 & 0 & 0 & 0 & 0 \end{pmatrix} \begin{pmatrix} S_4 \\ S_5 \\ U_{1,3} \\ U_{1,4} \\ U_{1,5} \\ U_{2,4} \\ U_{2,5} \\ U_{3,4} \\ U_{3,5} \\ U_{4,1} \\ U_{4,2} \\ U_{4,3} \end{pmatrix} = \begin{pmatrix} 1.7 \\ 11.6 \\ 2.3 \\ 10.4 \\ 5.8 \end{pmatrix} \tag{S5}$$

S2 Model convergence diagnostics

In this section we describe model diagnostic checks used to examine the convergence of the Markov Chains Monte Carlo (MCMC) sampling algorithm (No-U Turn Sampler of Hoffman and Gelman [2014]). We present trace plots for a selection of flow and change in stock variables in the aluminium model for illustration. Using the No-U-Turn Sampler, we sampled 2 independent chains of 12000 samples, with the first 2000 samples used for tuning and discarded from the final posterior samples. The purpose of the tuning samples are to give the Markov Chains iterations to converge to the target posterior distribution before extracting samples to approximate the posterior distribution. The traceplots in Figure S2 suggests both chains have converged to the same distribution. Furthermore, for both scenario A and scenario B of the aluminium model, the Gelman-Rubin diagnostic statistic (see for example Chapter 11 of Gelman et al. [2013]) for all flow and change in stock variables in the model have converged to 1.0 and no divergent samples were reported, indicating the posterior samples are reliable (Betancourt [2017]).

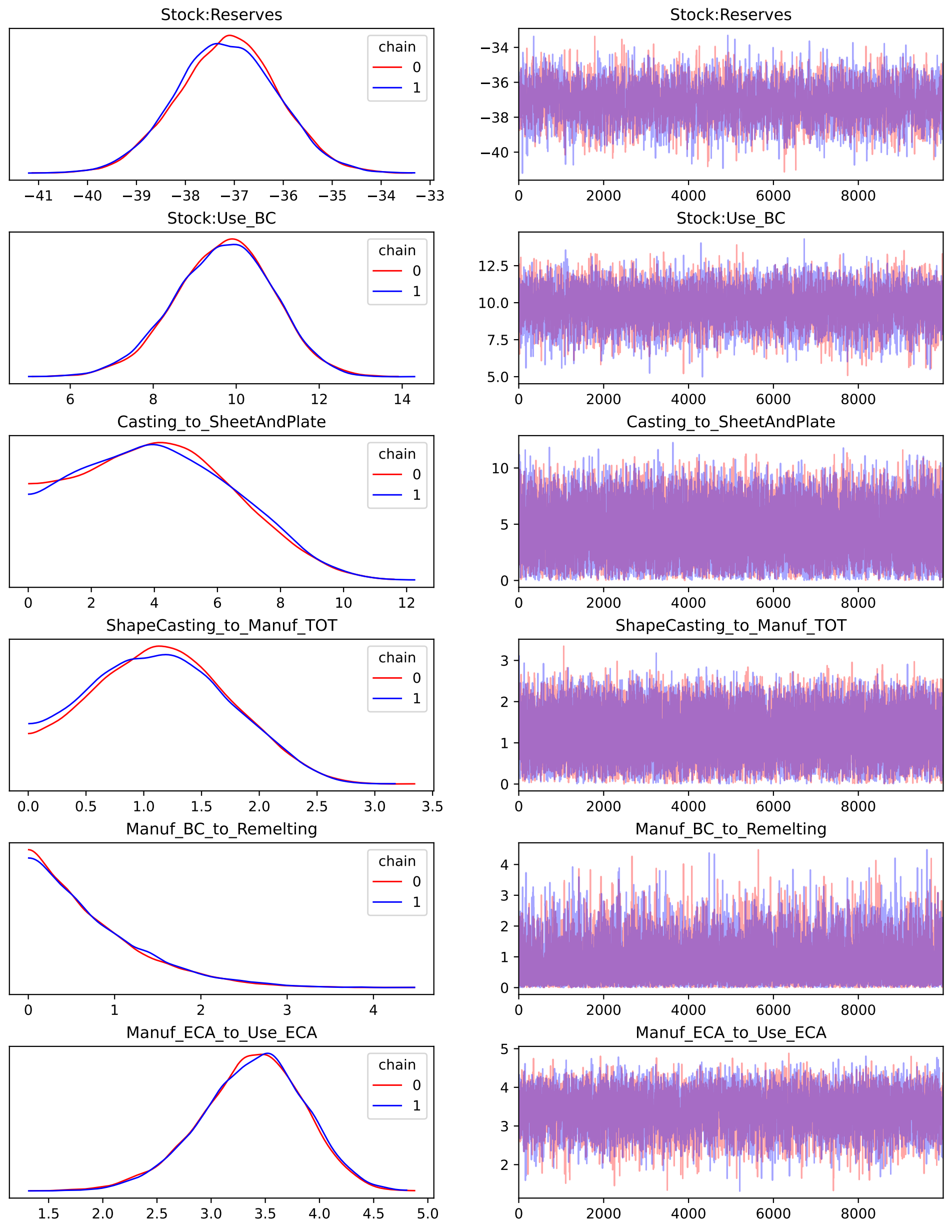

Figure S2: Traceplots for a selection of flow and change in stock variables in the aluminium model, scenario A.

S3 Posterior pairplots

Here we present a small selection of pairplots to illustrate correlation between the posterior distribution of flow variables. In particular, we display all the outflows of the 'Foil' process, which is a subprocess of semi-manufacturing. The outflow processes of 'Foil' are 'InternalRemelting', 'Manuf_TAU', 'Manuf_TOT', 'Manuf_POT', 'Manuf_CD'. Here one can see from the pairplots that it's very uncommon for the total of any two outflows to exceed around 6 Mt, which is consistent with the data which has the total outflow of 'Foil' at 6.4 Mt.

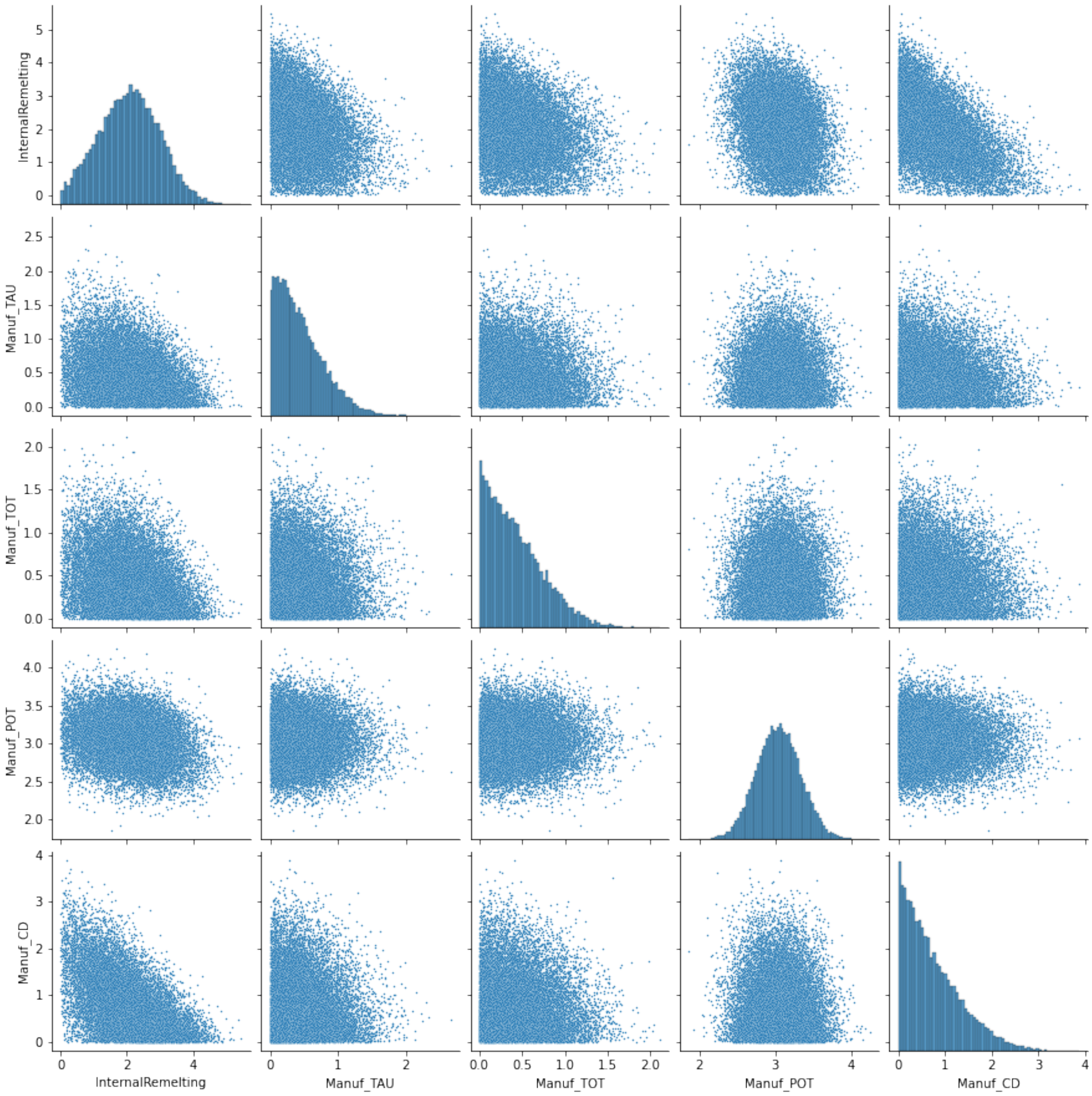

Figure S3: Pairplots for the outflows of the Foil process, scenario A. Axis units are in Mt.

S4 Detail of prior parameters for the aluminium model

In this section we give detail on the choice of prior parameters for the model evaluation of the aluminium dataset, A summary of the parameter values described in this section can be found in Table S1. Below we describe and motivate the choices:

The prior mode μ_i or $\mu_{j,k}$ of flow and change in stock variables respectively are chosen to be equal to the nearest power of 10 of the reported value when it is available. For example, the flow from 'Mining' to 'Refining' with a reported value 35.4 Mt is assigned a prior mode of $\mu_{1,2} = 10.0$ Mt. If the flow or change in stock variable has no reported value, it is assigned an uninformative prior mode of 1.0 Mt instead.

The prior standard deviation σ_i for the change in stock variables are chosen to be $\sigma_i = \max(\sqrt{40}|\mu_i|, 0.1)$, or 10.0 if there is no reported value for the flow or change in stock variable. The corresponding parameter $\sigma_{j,k}$ for the flow variables are chosen similarly. For example, $\sigma_{1,2} = \sqrt{40} * 10.0 = 63.25$ for the flow from 'Mining' to 'Refining'. This choice is motivated by when μ_i is specified to the correct order of magnitude, the true value of the variable is at most $4.5|\mu_i|$ away, and so σ_i is chosen on a similar scale but somewhat larger to ensure the prior is not over-confident. The maximum here is to ensure a minimum prior standard deviation of 0.1.

Note we are not advocating for this choice of prior distribution to be uniquely correct. The overall rationale of this choice for the prior is to assign a weakly informative prior distribution with a prior mode that captures the order of magnitude of the flow or change in stock, coupled with a relatively large prior variance, in order to reflect a realistic baseline of domain knowledge to aim for when conducting MFA.

For lack of better information, the standard deviation parameters for change in stock data noise $\tau_{i'}$ are chosen to be $\tau_{i'} = \max(0.1|Y_{i'}|, 0.1)$, where $Y_{i'}$ are the observed change in stock data. The corresponding parameters $\tau_{j'}$ for flow data noise is chosen similarly. In other words, the data standard deviation is equal to 10% of the observed data as in Lupton and Allwood [2018], but with a minimum of 0.1 to account for a minimum degree of uncertainty from rounding or other measurement errors. For the ratio data noise, the standard deviation parameters $\tau_{k'}$ are chosen with a somewhat smaller minimum $\tau_{k'} = \max(0.1Y_{k'}, 0.01)$, where $Y_{k'}$ is the observed ratio. While the choice of standard deviation parameters here is somewhat arbitrary, the posterior predictive checks performed on the aluminium model do not exhibit extreme Bayesian p-values (smaller than 0.05 or greater than 0.95), which suggests the standard deviation parameters chosen are reasonable. This also suggests one way to tune standard deviation parameters is to start with a small choice of standard deviation parameters (such as 10% of the observed data value), run the model, identify the data points which exhibit extreme Bayesian p-values in the posterior predictive checks, and increase the standard deviation parameter for those points and rerun the model until no extreme Bayesian p-values remain.

For the mass conservation conditions, a small constant standard deviation of $\tau_{l'} = 0.05$ is chosen for all mass balance conditions. Ideally these parameters should be chosen based on the modeller's confidence regarding epistemic uncertainty in the system, giving higher standard deviations to processes where there is more uncertainty in the system definition. Like with the standard deviation parameters for the data, posterior predictive checks can help select suitable parameter values, where the standard deviation parameters for mass balance conditions with extreme Bayesian p-values should be increased until the Bayesian p-values are no longer extreme. Without prior confidence of epistemic uncertainty in the system, we recommend starting with a small constant standard deviation for the mass conservation conditions, but not too small so that the mass conservation conditions are sufficiently relaxed to ensure the NUTS algorithm converges well. In this case it turned out the choice of $\tau_{l'} = 0.05$ did not produce any extreme Bayesian p-values, produces well converged posterior samples and well mass balanced flows (see subsequent section for more detail).

Parameter	Parameter prior value
μ_i	nearest power of 10 of reported value, or 1.0 if unavailable
$\mu_{j,k}$	nearest power of 10 of reported value, or 1.0 if unavailable
σ_i	$\max(\sqrt{40}\|\mu_i\|, 0.1)$, or 10.0 if reported value is unavailable
$\sigma_{j,k}$	$\max(\sqrt{40}\mu_{j,k}, 0.1)$, or 10.0 if reported value is unavailable
$\tau_{i'}$	$\max(0.1\|Y_{i'}\|, 0.1)$
$\tau_{j'}$	$\max(0.1Y_{j'}, 0.1)$
$\tau_{k'}$	$\max(0.1Y_{k'}, 0.01)$
$\tau_{l'}$	0.05

Table S1: Table of parameters for the aluminium model

S5 Assessment of posterior mass balance for the aluminium model

In this section we analyse how well the mass balance conditions are satisfied by the aluminium model, given the parameters chosen in the previous section, in particular the standard deviation parameters $\tau_{l'} = 0.05$ for the mass balance conditions. We examine the posterior distribution of the mass balance conditions to see how well they are centred around 0.

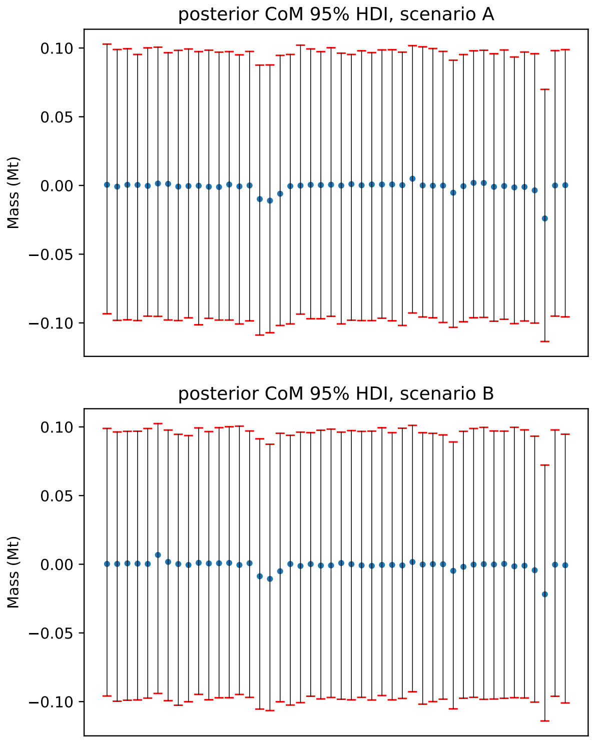

Figure S4: posterior HDI lengths for the mass conservation conditions. On the top we have scenario A and on the bottom we have scenario B. The 95% posterior highest density intervals are shown with a red bar and the posterior mean is shown with a blue dot

From the Figure above, it can be seen that the posterior mean of the mass balance conditions is very close to 0 in both cases, having a mean of -0.001 Mt for both scenario A and scenario B when averaged over all processes, which is orders of magnitudes smaller than the average posterior flow magnitude of around 2.3 Mt for both scenario A and B. This ensures that the posterior mean of the flow and stock change variables in the system, which is also the point estimate we report, is very well balanced. The individual posterior samples are somewhat less mass balanced, exhibiting a standard deviation of around 0.05 Mt for all processes. However, this is still a small level of mass imbalance and it is not necessary for every posterior sample to be perfectly mass balanced for the model to produce useful point estimation or uncertainty quantification.

S6 Zinc cycle simulation study

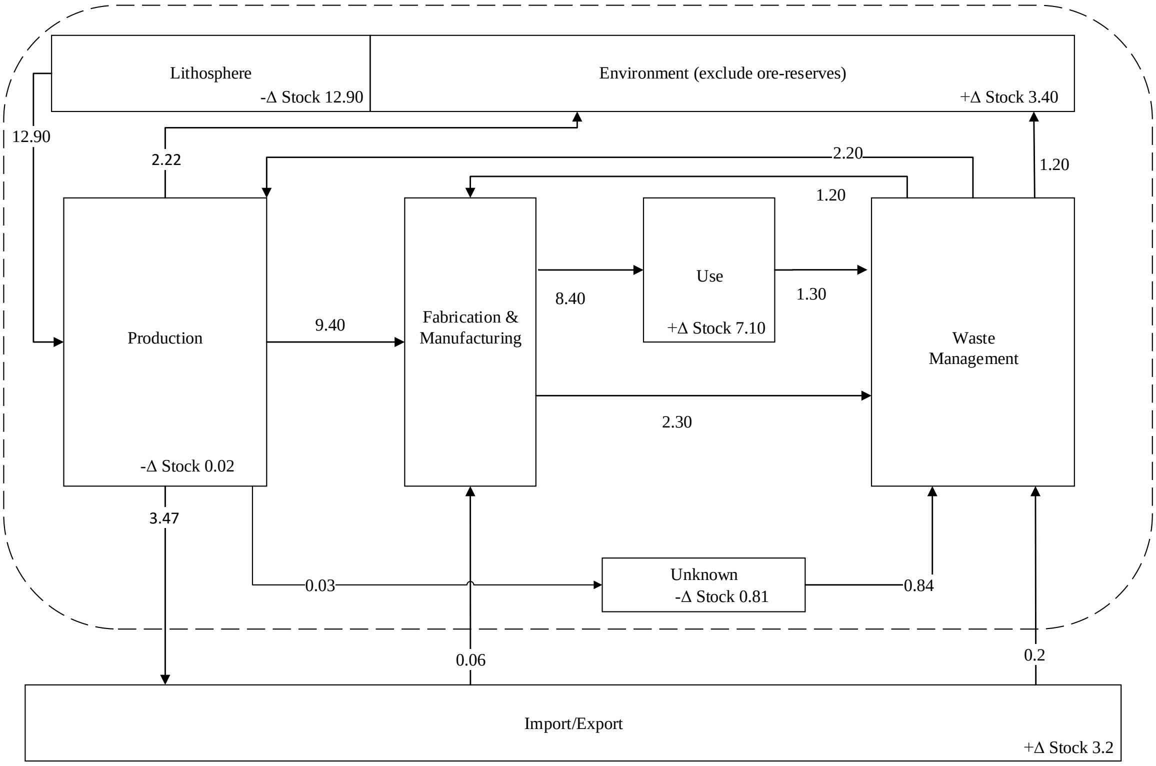

Figure S5: Zinc cycle for China, ca. 1994-1998, adapted from Figure 2d of Graedel et al. [2005]. The unit of mass for zinc is displayed in 10^5 metric tons per year. we explicitly define an 'Unknown' Process to account for flows with an unknown source or destination.

We conduct a simulation study to investigate the estimation accuracy and uncertainty quantification of our model. For this end we consider the zinc cycle in China, ca. 1994-1998, from Graedel et al. [2005]. The zinc cycle contains no missing data values, so for the purposes of this simulation, we treat the reported data values as the true parameter values of the flow and stock change variables. This allows us to assess our model by withholding some data from it and checking the model output against the true values of the stock change and flows. We examine how our model performs in terms of estimation accuracy and uncertainty quantification under different levels of prior knowledge, as data is incrementally added into the model. The zinc model considered is linear for ease of comparison with other models that require data to be in the form of a design matrix. However, we note that common MFA data types including flows, flow ratios and stock changes can be expressed under a linear model.

To assess estimation accuracy of the model, we calculate how the error of the model evolves as data is randomly added into the model one at a time, starting from no data, until the full dataset becomes available to the model. We consider two types of error, the root mean square error (rmse) and the maximum error, of the model's predictions compared to the reported values of the flow and change in stocks variables. The posterior mode is taken as the model prediction. To account for the random order in which data is added into the model, we repeat this process 50 times and calculate the average error (for both rmse and the maximum error) over the 50 runs for each possible data size from 0 to 20.

For comparison, we consider several alternate models. First, we consider an alternate Bayesian Gaussian model which has the advantage of a closed form solution, but carries the disadvantage of assigning significant probability to negative values for flow variables in the posterior distribution, which should be nonnegative as negative flows are not physically meaningful. Detail of the Gaussian model is

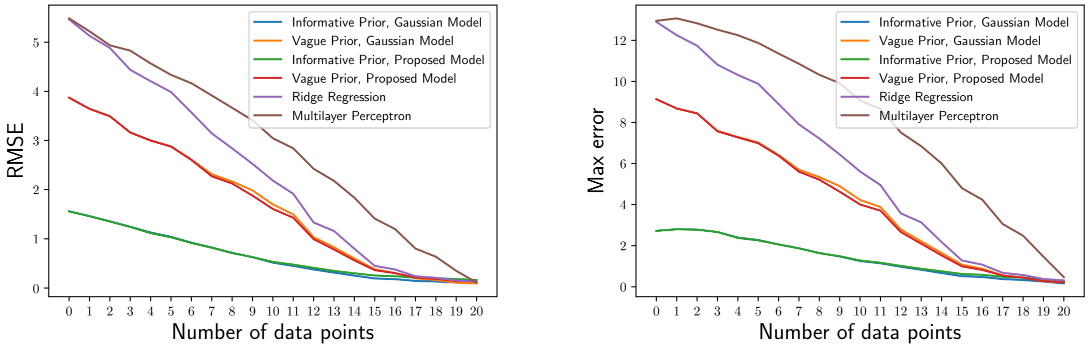

Figure S6: Zinc flow data error analysis: (left) Root mean squared error RMSE of the model plotted against number of data points available to the model, averaged over 50 runs or datasets. (right) Maximum error of the model plotted against the number of data points available to the model, averaged over 50 runs or datasets. For each error type, our model and the Gaussian model are considered under uninformative and informative priors. Ridge regression is provided as a non Bayesian baseline.

provided in Section S8. In addition, we also investigate the effect of different levels of prior knowledge has on the error. We utilise two different levels of prior knowledge, the first is an 'uninformative' prior which sets the prior mode of all variables to the average absolute value of the full data, and the correct sign for change in stock variables. This intuitively represents a prior that knows the average order of magnitude of the system but has little visibility on the individual magnitude of each flow or change in stock. The second is a 'weakly informative' prior which sets the prior mode of each flow and change in stock variable to the nearest power of 10 of the true value. This intuitively represents a prior that has knowledge of each flow and change in stock variable to the nearest order of magnitude. We also provide results from non Bayesian regression models for comparison, specifically ridge regression and multilayer perceptron (see e.g. Hastie et al. [2001]). For each run, both algorithms are trained on the same datasets as the proposed and Gaussian model, and outputs a prediction on the vector of all flows and change in stock variables. While these models do not have a principled way of incorporating prior information, one can nevertheless train those models on the data and use them to estimate the value of all flow and change in stock variables in a system, which makes a comparison with Bayesian MFA models meaningful.

Figure S6 shows the results of our analysis, where the rmse and maximum error of the model are plotted against the number of data points available to the model, averaged over 50 runs. For ridge regression and multilayer perceptron, the starting rmse at no data is around 5.4, whereas for the uninformative prior it is around 4.0 and for the weakly informative prior around 1.5. As data is added, all models exhibit the trend of monotonically decreasing error, but the proposed and Gaussian models exhibit noticeably lower error until almost all the data becomes available to the model, indicating incorporating prior information leads to more accurate estimates. Notably, it takes around 10 data points for the uninformative prior models and 12 data points for ridge regression to achieve the starting rsme (around 1.5) of the weakly informative prior model. Therefore, one can consider only knowing the order of magnitude of each flow or change in stock to be roughly equivalent to 10 data points in terms of rmse in this model, demonstrating the utility of being able to incorporate knowledge via a Bayesian prior in such an underdetermined system. For the Gaussian model, theoretical bounds on the mean square error provided in Section S8 also support the trend exhibited in Figure S6, and the intuition that having an informative prior can greatly increase the model's accuracy for MFA problems when there is a shortage of data but available expert domain knowledge.

To assess uncertainty quantification of the posterior distribution, we examine whether the 95% posterior marginal highest density intervals (HDIs) of our model have similar level of frequentist coverage, i.e. form correct confidence intervals. Unlike credible intervals which have a designated posterior probability of containing the unknown parameter, confidence intervals are constructed such that upon repeated experiments (i.e. generate samples of data), a designated proportion (known as coverage) of the confidence intervals constructed will contain the true value of the unknown parameter. Bayesian credible intervals are in general not guaranteed to have good coverage, particularly in high-dimensional settings that are

typical in MFA, but nevertheless it is desirable for them to have good coverage. Coverage is a property of the model procedure and does not require the repeated experiments to be the same experiment or dataset. It is therefore desirable for uncertainty quantification generated by a model procedure to contain the true value of the unknown parameter most of the time, over many runs for possibly different datasets. We investigate the coverage of our model's credible intervals empirically in order to understand whether they can be used reliably as confidence sets.

The high dimensional nature of MFA models means the dimension of parameter space p (corresponding to the number of flow and change in stock variables in the model) is usually greater or similar in size compared to the number of available data n, and n is typically not large in magnitude. Therefore, results which guarantees asymptotic frequentist coverage of credible regions, such as the Bernstein-von Mises theorem (see e.g. Vaart [1998]), may not apply in MFA problems. We therefore study in simulations the frequentist coverage of our model's posterior credible intervals, in an effort to understand whether these can be used as reliable confidence intervals. Specifically, we sample 300 sets of the zinc dataset $\boldsymbol{Y}_1, \boldsymbol{Y}_2, \ldots, \boldsymbol{Y}_{300}$ from the model likelihood $p(\boldsymbol{Y}|\boldsymbol{\theta}_0)$, where we take the 'true values' $\boldsymbol{\theta}_0$ to be the originally reported flow and change in stock values reported in Graedel et al. [2005] . For each dataset sampled, we add data to the model in batches of 5 datapoints at a time (in the order listed from top to bottom in Table S2), and calculate the posterior marginal HDIs to see if they contain the true value for each dataset size $n = 5, 10, 15, 20$. The frequencies at which the posterior marginal HDIs contain the true value over the 300 runs, known as the coverage probability, are reported in Table S2.

	Coverage probability(%), average width of 95% HDI							
	Weakly informative prior				Uninformative prior			
Variable Name	5 data	10 data	15 data	20 data	5 data	10 data	15 data	20 data
ΔStock: Use	95.3, 3.60	96.3, 3.44	98.0, 3.16	96.3, 3.15	96.0, 3.76	95.3, 3.63	97.7, 3.23	95.7, 3.21
Production to ImportExport	90.7, 3.56	96.3, 3.40	96.7, 3.41	98.0, 2.92	91.3, 3.78	95.7, 3.65	97.7, 3.60	96.0, 3.15
ΔStock: Environment	98.0, 3.50	97.0, 3.35	97.3, 3.35	98.7, 3.18	95.0, 3.66	97.3, 3.44	96.0, 3.46	98.3, 3.25
WM to Production	94.7, 3.39	96.0, 3.15	96.7, 3.12	93.0, 3.02	94.7, 3.54	94.0, 3.47	94.7, 3.35	91.3, 3.24
ImportExport to WM	100.0, 0.83	100.0, 0.82	100.0, 0.82	100.0, 0.79	96.0, 2.29	99.3, 2.16	97.0, 2.12	98.7, 1.82
Unknown to WM	100.0, 5.07	99.0, 2.48	99.3, 2.18	100.0, 2.19	100.0, 8.52	99.0, 2.53	98.0, 2.42	97.7, 2.48
Lithosphere to Production	100.0, 7.73	96.3, 3.37	95.3, 3.38	93.7, 2.87	0.00, 11.3	96.0, 3.63	94.7, 3.55	94.7, 2.97
ΔStock: Production	100.0, 0.39	100.0, 0.39	100.0, 0.39	100.0, 0.39	100.0, 16.1	95.0, 3.67	95.3, 3.59	96.0, 3.50
Production to F&M	100.0, 7.69	94.7, 3.32	99.0, 3.19	93.7, 3.06	3.33, 8.25	89.0, 3.55	91.7, 3.39	87.0, 3.21
WM to Environment	100.0, 4.19	98.0, 2.69	98.7, 2.50	99.0, 2.55	100.0, 4.88	96.3, 2.98	97.3, 2.72	95.7, 2.72
F&M to Use	100.0, 7.54	99.7, 7.20	96.3, 3.03	93.0, 2.95	100.0, 9.55	100.0, 9.57	96.3, 3.14	93.3, 3.02
ΔStock: Unknown	100.0, 6.58	100.0, 4.62	100.0, 2.97	98.7, 2.97	100.0, 13.3	100.0, 7.20	98.0, 3.16	99.3, 3.16
Production to Unknown	100.0, 0.20	100.0, 0.20	100.0, 0.20	100.0, 0.20	100.0, 9.31	97.7, 4.90	93.7, 2.03	96.7, 2.01
F&M to WM	100.0, 6.44	100.0, 6.52	93.3, 3.30	94.3, 3.12	100.0, 10.6	100.0, 11.7	93.3, 3.45	93.7, 3.21
Use to WM	100.0, 5.91	100.0, 5.93	98.3, 2.60	98.0, 2.54	100.0, 7.32	100.0, 7.71	98.0, 2.63	98.7, 2.57
WM to F&M	100.0, 7.79	100.0, 7.29	100.0, 3.79	98.3, 2.63	95.0, 14.1	100.0, 12.4	100.0, 4.16	97.7, 2.74
ΔStock: ImportExport	97.0, 5.21	99.3, 5.10	99.7, 5.10	96.0, 3.09	84.0, 9.63	90.3, 7.30	95.3, 6.55	93.0, 3.26
ΔStock: Lithosphere	100.0, 8.21	99.0, 4.95	99.3, 4.95	95.3, 3.04	0.00, 12.2	99.0, 5.24	98.7, 5.18	95.7, 3.11
ImportExport to F&M	100.0, 0.86	100.0, 0.85	100.0, 0.82	100.0, 0.79	100.0, 7.19	100.0, 4.39	100.0, 3.17	98.0, 1.80
Production to Environment	100.0, 3.77	100.0, 3.74	99.3, 3.92	95.0, 2.90	100.0, 4.20	99.0, 3.74	99.7, 4.14	96.0, 2.97

Table S2: Table of coverage probability and average width of 95% highest density intervals (HDI) of the marginal posterior distributions of all flow and change in stock variables in the zinc data under different amounts of data and prior information, computed over 300 runs. Data was added in batches of 5 to the model in the order that they are listed in this table (from top to bottom).

From Table S2, it can be seen that the 95% HDI possess similar levels of coverage probability in most cases. The only notable exception being the uninformative prior at 5 data points, where the coverage probability are close to 0 for the flow variables 'Lithosphere to Production' and 'Production to F& M', and the change in stock variable for 'Lithosphere'. This is likely caused by the first 5 data points not containing any data on the 'Lithosphere' process, as well as containing little data on 'Production', so the model was unable deduce the true magnitude of how much material was taken from the 'Lithosphere' with an uninformative prior. On the other hand, the weakly informative prior does not possess this issue as the prior has sufficient coverage over the true value. In addition, the average width of the 95% HDI are generally lower for the weakly informative prior until data for that variable becomes available, which is expected from a more informative prior.

S7 Detail of parameters for the zinc models

In this section we give detail on the choice of model parameters for all models used for the zinc case study, including how the prior parameters were chosen. Recall that we considered two different priors for the zinc model, an uninformative prior and a weakly informative prior. For the uninformative prior, the prior modes μ_i of the change in stock variables are chosen to be 3.6575 (measured in 10^5 tonnes/Yr), equal to the mean of the absolute value of all the stock change and flow variables in the data, to represent a prior that only has a rough knowledge of the average order of magnitude of the system, but not the order of magnitude of each individual flow or change in stock. For weakly informative prior, μ_i are chosen the same way as the aluminium case, where the prior mode is set to the nearest power of 10 of the 'true' reported value. The prior standard deviation parameters σ_i are chosen to be a constant $\sqrt{40}$ in the uninformative case, and $\sigma_i = \max(\min(4|\mu_i|, 4), 0.1)$ in the weakly informative case. Like in the aluminium model, σ_i for the weakly informative prior is chosen at a similar scale compared to $4.5|\mu_i|$ but somewhat smaller and capped at a maximum of 4.0 to reflect the fact that the size of flows are relatively smaller in the zinc data (when measured in 10^5 tonnes/Yr, compared to the aluminium data which is measured in Mt). The corresponding prior parameters for the flow variables $\mu_{j,k}$ and $\sigma_{j,k}$ are chosen similarly.

The noise standard deviation parameters of the observed data is chosen to be a constant $\tau = 1.0$ throughout for all data, and similarly for the mass conservation conditions. Here the noise parameters are chosen to be somewhat larger than the aluminium model to speed up the computation of the No-U-Turn Sampler MCMC algorithm, as the model needed to be computed 2400 times over the varying degrees of prior knowledge and available data to generate the coverage probability results in Table S2. In particular, small values of the noise parameter induces high curvature which makes the sampling algorithm take longer to explore the posterior distribution. Similarly, for assessing point estimation (root mean squared error and maximum error) of the model on the zinc data, the posterior mode of the model is estimated directly via optimisation for faster computational speed, rather than through approximating the entire posterior distribution using MCMC.

Parameter	Parameter prior value (weakly informative prior)	Parameter prior value (uninformative prior)
μ_i	nearest power of 10 of reported value	± 3.6575 (sign depending if the change in stock is positive or negative)
$\mu_{j,k}$	nearest power of 10 of reported value	3.6575
σ_i	$\max(\min(4\|\mu_i\|, 4), 0.1)$	$\sqrt{40}$
$\sigma_{j,k}$	$\max(\min(4\mu_{j,k}, 4), 0.1)$	$\sqrt{40}$
τ	1.0	1.0

Table S3: Table of parameters for the zinc model

The ridge regression model was trained with a regularization parameter of $\lambda = 1.0/40.0 = 0.025$, which was chosen to match the prior variance and noise standard deviation in the uninformative prior case for the Gaussian model. To see this, note the solution of the ridge regression is equal to the posterior mode of a model with prior $\boldsymbol{\theta} \sim \mathcal{N}(0, \sigma^2 I)$ and likelihood $\boldsymbol{Y}|\boldsymbol{\theta} \sim \mathcal{N}(X\boldsymbol{\theta}, \tau^2 I)$, and the regularization parameter can be shown to be equal to τ^2/σ^2. So effectively the ridge regression solution is similar to the uninformative prior case, except the prior mean is set to 0 instead which is arguably less informative.

The multilayer perceptron regressor was trained with the default settings in the sklearn Python library, except with the maximum number of iterations increased to 1000. In particular the default loss function is the squared loss and default number of hidden layers equal to 100.

S8 Gaussian model

For model comparison purposes and to gain intuition into how Bayesian models perform for MFA problems, it is useful to examine a model with simplified assumptions. In particular, we consider a Gaussian model, where both the prior and the likelihood function are assumed to be normally distributed, and the data is assumed to be linear in terms of $\boldsymbol{\theta}$ (recall from the Methods section in the main text of the paper that the most common forms of MFA data can be expressed linearly in $\boldsymbol{\theta}$):

$$\boldsymbol{\theta} \sim \mathcal{N}(\boldsymbol{\mu}, \Sigma) \tag{S6}$$
$$Y_i|\boldsymbol{\theta} \sim \mathcal{N}(\boldsymbol{x_i}^\top \boldsymbol{\theta}, \tau_i^2) \tag{S7}$$

where $\boldsymbol{\mu}$ is the prior mean and Σ is the (positive definite) prior covariance matrix. This gives a mathematically convenient *conjugate* posterior that is also normally distributed, with its posterior mean $\boldsymbol{\mu}^n$ and covariance Σ^n available in closed form:

$$\boldsymbol{\mu}^n = \boldsymbol{\mu} + \Sigma X^\top (X\Sigma X^\top + T)^{-1}(\boldsymbol{Y} - X\boldsymbol{\mu}) \tag{S8}$$
$$\Sigma^n = \Sigma - \Sigma X^\top (X\Sigma X^\top + T)^{-1} X\Sigma \tag{S9}$$

where T is a n by n diagonal matrix with entries τ_i^2, $1 \leq i \leq n$. An advantage of the Gaussian model is that it allows the posterior mean and covariance to be calculated directly without potentially computationally intensive MCMC algorithms. This makes the Gaussian model an easy to compute baseline to compare with more complicated Bayesian models. Furthermore, owing to the closed form of the posterior, we can bound the mean squared error (MSE) between the posterior mean $\boldsymbol{\mu}^n$ and the true parameter (stock changes and flows) values $\boldsymbol{\theta}^*$. A discussion on this is provided below, which gives some intuition why Bayesian methods can perform better than non Bayesian methods in the low data setting $n \ll p$.

While computationally convenient, an disadvantage with the Gaussian conjugate model is that the posterior distributions for flow variables can contain non negligible probability on negative values when there is insufficient data or uninformative priors, which is not physically meaningful as flow quantities should be strictly positive, making it not fully Bayesian in this sense. While the posterior mean of the Gaussian model can perform adequately in terms of point estimation, we do not recommend it for uncertainty quantification without very informative priors.

Nevertheless, the Gaussian model usefully gives some intuition into why Bayesian methods perform better than non Bayesian methods in the low data setting $n \ll p$. Below we provide some theoretical analysis for the mean squared error of the Gaussian model.

Owing to the closed form of the posterior of the Gaussian model, we can bound the mean squared error (MSE) between the posterior mean $\boldsymbol{\mu}^n$ and the true parameter (stocks changes and flows) values $\boldsymbol{\theta}^*$ as follows:

Theorem 1. *Let $\tau_i^2 = \tau^2$, in other words $T = \tau^2 I$, I the identity matrix. The mean squared error between the posterior mean of the Gaussian posterior $\boldsymbol{\mu}^n$ and the true stock change and flow values $\boldsymbol{\theta}^*$ can be bounded above in the following way:*

$$\begin{aligned}
&\mathbb{E}(\|\boldsymbol{\theta}^* - \boldsymbol{\mu}^n\|_2^2) = \mathbb{E}(\sum_{j=1}^{p}(\theta_j^* - \mu_j^n)^2) \\
&\leq \mathrm{Tr}(\Sigma^{-1/2}(\boldsymbol{\theta}^* - \boldsymbol{\mu})(\boldsymbol{\theta}^* - \boldsymbol{\mu})^\top \Sigma^{-1/2})\lambda_1(p - n + \sum_{j=1}^{n}\frac{\tau^4}{(d_j^2 + \tau^2)^2}) + \tau^2 \sum_{j=1}^{n}\frac{\lambda_1 d_j^2}{(d_j^2 + \tau^2)^2}
\end{aligned} \tag{S10}$$

where $\lambda_1 \geq \lambda_2 \geq \cdots \geq \lambda_p > 0$ are the eigenvalues of Σ in descending order, and $0 \leq d_1 \leq d_2 \leq \cdots \leq d_n$ are the singular values of $X\Sigma^{1/2}$ in ascending order.

The bound in Equation (S10) helps to give some insight into how the mean squared error of the model evolves as more data is added. This is perhaps easiest to see in the case when τ is near 0 (meaning the

data is almost noiseless) and $0 \leq \tau \ll d_1$, which leaves

$$\mathrm{Tr}(\Sigma^{-1/2}(\boldsymbol{\theta}^* - \boldsymbol{\mu})(\boldsymbol{\theta}^* - \boldsymbol{\mu})^\top \Sigma^{-1/2})\lambda_1(p-n)$$

as the dominant term. The $\mathrm{Tr}(\Sigma^{-1/2}(\boldsymbol{\theta}^* - \boldsymbol{\mu})(\boldsymbol{\theta}^* - \boldsymbol{\mu})^\top \Sigma^{-1/2})$ part of this term can be interpreted as a measurement of how close the prior mean is to the true values of the parameters (it is 0 when $\boldsymbol{\theta}^* = \boldsymbol{\mu}$) and does not depend on the data, while the factor $\lambda_1(p-n)$ decreases as n increases, meaning as more data is added. This indicates that in a high dimensional setting $n \ll p$ which is inherent in MFA studies, the availability of informative priors is key to obtaining more accurate estimates of the true stock change and flow values.

Furthermore, in the case when $\Sigma = \lambda_1 I$, Equation (S10) can be directly compared with the ridge regression case. Since ridge regression is equivalent to the case when $\boldsymbol{\mu} = \mathbf{0}$ and $\Sigma = \lambda_1 I$, Equation (S10) is smaller for the Gaussian model compared to ridge regression whenever $\mathrm{Tr}((\boldsymbol{\theta}^* - \boldsymbol{\mu})(\boldsymbol{\theta}^* - \boldsymbol{\mu})^\top) = \|\boldsymbol{\theta}^* - \boldsymbol{\mu}\|_2^2$ is smaller than $\|\boldsymbol{\theta}^*\|_2^2$. In other words whenever the prior mean $\boldsymbol{\mu}$ is closer to the true value in (L2) distance than the zero vector $\mathbf{0}$, which is consistent with the simulations presented in Section S6.

Proof of Theorem 1. Recall that the posterior mean of the conjugate Gaussian model can be written as:

$$\boldsymbol{\mu}^n = \boldsymbol{\mu} + \Sigma X^\top (X\Sigma X^\top + \tau^2 I)^{-1}(X(\boldsymbol{\theta}^* - \boldsymbol{\mu}) + \boldsymbol{\epsilon})$$

This can be derived from looking at the joint distribution $[\boldsymbol{\theta}, X\boldsymbol{\theta} + \boldsymbol{\epsilon}]^\top$ and using standard Gaussian conditioning formulae:

$$\begin{bmatrix} \boldsymbol{\theta} \\ X\boldsymbol{\theta} + \boldsymbol{\epsilon} \end{bmatrix} \sim \mathcal{N}\left(\begin{bmatrix} \boldsymbol{\mu} \\ X\boldsymbol{\mu} \end{bmatrix}, \begin{bmatrix} \Sigma & \Sigma X^\top \\ X\Sigma & X\Sigma X^\top + \tau^2 I \end{bmatrix} \right) \tag{S11}$$

Suppose $dim(X) = (n, p)$, $dim(\Sigma) = (p, p)$, $dim(\boldsymbol{\mu}) = dim(\boldsymbol{\theta}^*) = (p, 1)$, $dim(\boldsymbol{\epsilon}) = (n, 1)$, and we'll assume $n < p$, since material flow analysis typically has less data than parameters. Assume Σ is symmetric positive definite and X has full rank. The mean squared error (MSE) between the posterior mean $\boldsymbol{\mu}^n$ and the true value $\boldsymbol{\theta}^*$ is equal to:

$$\begin{aligned} MSE &= \mathbb{E}\|\boldsymbol{\theta}^* - \boldsymbol{\mu} - \Sigma X^\top (X\Sigma X^\top + \tau^2 I)^{-1}(X(\boldsymbol{\theta}^* - \boldsymbol{\mu}) + \boldsymbol{\epsilon})\|_2^2 \\ &= \mathbb{E}(\boldsymbol{\beta} - \Sigma X^\top (X\Sigma X^\top + \tau^2 I)^{-1}(X\boldsymbol{\beta} + \boldsymbol{\epsilon}))^\top (\boldsymbol{\beta} - \Sigma X^\top (X\Sigma X^\top + \tau^2 I)^{-1}(X\boldsymbol{\beta} + \boldsymbol{\epsilon})) \\ &= \mathbb{E}(\boldsymbol{\beta}^\top (I - \Sigma X^\top (X\Sigma X^\top + \tau^2 I)^{-1} X)^\top (I - \Sigma X^\top (X\Sigma X^\top + \tau^2 I)^{-1} X)\boldsymbol{\beta}) \\ &+ \mathbb{E}(\boldsymbol{\epsilon}^\top (\Sigma X^\top (X\Sigma X^\top + \tau^2 I)^{-1})^\top \Sigma X^\top (X\Sigma X^\top + \tau^2 I)^{-1} \boldsymbol{\epsilon}) \end{aligned}$$

Where $\boldsymbol{\beta} = \boldsymbol{\theta}^* - \boldsymbol{\mu}$. At this point we decompose the MSE into the bias and variance terms. Let

$$MSE_1 = \mathbb{E}(\boldsymbol{\beta}^\top (I - \Sigma X^\top (X\Sigma X^\top + \tau^2 I)^{-1} X)^\top (I - \Sigma X^\top (X\Sigma X^\top + \tau^2 I)^{-1} X)\boldsymbol{\beta})$$

denote the bias term. and

$$MSE_2 = \mathbb{E}(\boldsymbol{\epsilon}^\top (\Sigma X^\top (X\Sigma X^\top + \tau^2 I)^{-1})^\top \Sigma X^\top (X\Sigma X^\top + \tau^2 I)^{-1} \boldsymbol{\epsilon})$$

denote the variance term.

For the bias term, using the fact that the trace of a scalar is equal to itself and the cyclic property of trace, we have:

$$\begin{aligned} MSE_1 &= \mathrm{Tr}(\boldsymbol{\beta}^\top (I - \Sigma X^\top (X\Sigma X^\top + \tau^2 I)^{-1} X)^\top (I - \Sigma X^\top (X\Sigma X^\top + \tau^2 I)^{-1} X)\boldsymbol{\beta}) \\ &= \mathrm{Tr}(\boldsymbol{\beta}\boldsymbol{\beta}^\top (I - \Sigma X^\top (X\Sigma X^\top + \tau^2 I)^{-1} X)^\top (I - \Sigma X^\top (X\Sigma X^\top + \tau^2 I)^{-1} X)) \\ &= \mathrm{Tr}(\boldsymbol{\beta}\boldsymbol{\beta}^\top \Sigma^{-1/2}\Sigma^{1/2}(I - X^\top (X\Sigma X^\top + \tau^2 I)^{-1} X\Sigma)\Sigma^{-1/2}\Sigma\Sigma^{-1/2}(I - \Sigma X^\top (X\Sigma X^\top + \tau^2 I)^{-1} X)\Sigma^{1/2}\Sigma^{-1/2}) \\ &= \mathrm{Tr}(\Sigma^{-1/2}\boldsymbol{\beta}\boldsymbol{\beta}^\top \Sigma^{-1/2}(I - \Sigma^{1/2} X^\top (X\Sigma X^\top + \tau^2 I)^{-1} X\Sigma^{1/2})\Sigma(I - \Sigma^{1/2} X^\top (X\Sigma X^\top + \tau^2 I)^{-1} X\Sigma^{1/2})) \end{aligned}$$

Notice $\Pi_\tau = \Sigma^{1/2}X^\top(X\Sigma X^\top + \tau^2 I)^{-1}X\Sigma^{1/2}$ is symmetric. Similarly $I - \Pi_\tau$ is symmetric and so $(I - \Pi_\tau)^2$ is positive semidefinite.

Let $X\Sigma^{1/2} = UDV^\top$ be the singular value decomposition of $X\Sigma^{1/2}$, where $d_1 \leq d_2 \leq \cdots \leq d_n$ are the diagonal elements of D (also known as the singular values of $X\Sigma^{1/2}$) arranged in ascending order, then we have:

$$\Pi_\tau = VD^\top U^\top(UDD^\top U^\top + \tau^2 I)^{-1}UDV^\top = VD^\top(DD^\top + \tau^2 I)^{-1}DV^\top$$

which has the same eigenvalues as $D^\top(DD^\top + \tau^2 I)^{-1}D$ by similarity (Horn and Johnson [1985]), which are $d_i^2/(d_i^2 + \tau^2)$ and 0 (repeated $p - n$ times). Likewise the eigenvalues of $I - \Pi_\tau$ are therefore 1 (repeated $p - n$ times) and $\tau^2/(d_i^2 + \tau^2)$

returning to the bias term, we have:

$$\begin{aligned}
MSE_1 &= \operatorname{Tr}(\Sigma^{-1/2}\boldsymbol{\beta}\boldsymbol{\beta}^\top\Sigma^{-1/2}(I - \Pi_\tau)\Sigma(I - \Pi_\tau)) \\
&\leq \operatorname{Tr}(\Sigma^{-1/2}\boldsymbol{\beta}\boldsymbol{\beta}^\top\Sigma^{-1/2})\operatorname{Tr}((I - \Pi_\tau)^2\Sigma) \\
&\leq \operatorname{Tr}(\Sigma^{-1/2}\boldsymbol{\beta}\boldsymbol{\beta}^\top\Sigma^{-1/2})(\sum_{j=1}^{p-n}\lambda_j + \sum_{j=1}^{n}\lambda_{p-n+j}\tau^4/(d_j^2 + \tau^2)^2) \\
&\leq \operatorname{Tr}(\Sigma^{-1/2}\boldsymbol{\beta}\boldsymbol{\beta}^\top\Sigma^{-1/2})\lambda_1(p - n + \sum_{j=1}^{n}\tau^4/(d_j^2 + \tau^2)^2) \qquad \text{(S12)}
\end{aligned}$$

Where we used Von Neumann's trace inequality (Mirsky [1975]) in the penultimate line, on the matrices $(I - \Pi_\tau)^2$ and Σ. So provided τ^2 is small compared to the d_j^2, MSE_1 decreases approximately linearly in n, as n increases to p.

For the variance term MSE_2, we once again use the singular value decomposition $X\Sigma^{1/2} = UDV^\top$:

$$\begin{aligned}
MSE_2 &= \operatorname{Tr}(\mathbb{E}(\boldsymbol{\epsilon}^\top(\Sigma X^\top(X\Sigma X^\top + \tau^2 I)^{-1})^\top\Sigma X^\top(X\Sigma X^\top + \tau^2 I)^{-1}\boldsymbol{\epsilon})) \\
&= \operatorname{Tr}(\mathbb{E}(\boldsymbol{\epsilon}\boldsymbol{\epsilon}^\top(\Sigma X^\top(X\Sigma X^\top + \tau^2 I)^{-1})^\top\Sigma X^\top(X\Sigma X^\top + \tau^2 I)^{-1})) \\
&= \tau^2\operatorname{Tr}((X\Sigma X^\top + \tau^2 I)^{-2}X\Sigma^2 X^\top) \\
&= \tau^2\operatorname{Tr}((UDD^\top U^\top + \tau^2 I)^{-2}UDV^\top\Sigma VD^\top U^\top) \\
&= \tau^2\operatorname{Tr}(U^\top(UDD^\top U^\top + \tau^2 I)^{-1}UU^\top(UDD^\top U^\top + \tau^2 I)^{-1}UDV^\top\Sigma VD^\top) \\
&= \tau^2\operatorname{Tr}(VD^\top(DD^\top + \tau^2 I)^{-2}DV^\top\Sigma) \\
&\leq \tau^2\sum_{j=1}^{n}\lambda_1 d_j^2/(d_j^2 + \tau^2)^2
\end{aligned}$$

Where the last line again follows from Von Neumann's trace inequality, on the matrices $VD^\top(DD^\top + \tau^2 I)^{-2}DV^\top$ and Σ.

□

S9 Discussion of model parametrisation

In this section we discuss some additional limitations with the parametrisation of Gottschalk et al. [2010] which led us to use a mass based parametrisation. These limitations do not apply in all MFA systems but could potentially be problematic in some. In order to discuss them we provide a summary of the parametrisation: let $U_{i,j}$ denote the value of the flow from process i to process j. Let $z_i = \sum_k U_{i,k}$ denote the total outflow of process i, and $\phi_{i,j} = U_{i,j}/z_i$ be the transfer coefficient for the flow from process i to process j, equal to the fraction of the total outflow z_i of process i. In addition, an optional external flow q_i is allowed for each process i. Applying conservation of mass at process i, we obtain:

$$q_i + \sum_j U_{j,i} = \sum_k U_{i,k} \tag{S13}$$

which can be rewritten as

$$q_i + \sum_j z_j \phi_{j,i} = z_i \tag{S14}$$

the conservation of mass equations S14 can therefore be written in the matrix form

$$(I - \Phi^\top)\boldsymbol{z} = \boldsymbol{q} \tag{S15}$$

where Φ is a matrix with elements $\phi_{i,j}$. Lupton and Allwood [2018] and Gottschalk et al. [2010] then notes that this equation can be inverted to give $(I - \Phi^\top)^{-1}\boldsymbol{q} = \boldsymbol{z}$, the vector of the total outflow of every process, which can then be used to retrieve each individual flow variables $U_{i,j}$ via the relationship $U_{i,j} = \phi_{i,j} z_i$. Using this, Lupton and Allwood [2018] and Gottschalk et al. [2010] assigns priors on the transfer coefficients $\phi_{i,j}$ and the external flow variables q_i, which gives an implicit prior over the flow variables $U_{i,j}$.

The first limitation is that it is unclear if $I - \Phi^\top$ is always invertible. From $\phi_{i,j} = U_{i,j}/\sum_k U_{i,k}$ we must have $\sum_k \phi_{i,k} = 1$, which means the matrix Φ has each row summing to 1 (making it a stochastic matrix), which implies it must have eigenvalue 1 (this can be easily shown by checking the vector with entries all equal to 1 is an eigenvector). $I - \Phi^\top$ therefore must have 0 as an eigenvalue and is not invertible.

It appears the way this issue is currently handled is to have at least one process in the system that has no outflows (see for example Figure 1 of Dong et al. [2023], which has processes 6,7,8 and 9 without outflows), essentially fixing one or more rows of Φ to equal to the $\mathbf{0}$ vector so that it is no longer a stochastic matrix. However, it is unclear if a process that has no outflows always exists (for example Figure 2 of Mannan and Al-Ghamdi [2020]) and such processes must be interpretable as not mass balanced (e.g. a stock or outflow of some kind).

Additionally, if $\boldsymbol{q}$ is no longer nonnegative (e.g. if a stock change, external outflow, or mass balance relaxation term is added to the right hand side of S15) and assuming $I - \Phi^\top$ is invertible, it will cause the total flow output variables $\boldsymbol{z} = (I - \Phi^\top)^{-1}\boldsymbol{q}$ and by extension the flow variables $U_{i,j}$ to no longer be nonnegative. However, flow variables should be nonnegative to be physically meaningful. To see this, note that $(I - \Phi^\top)^{-1}$ has Taylor expansion $\sum_{m=0}^{\infty}(\Phi^\top)^m$, which is a limit of a sum of matrices with nonnegative entries, so the entries of $(I - \Phi^\top)^{-1}$ must all be nonnegative. Therefore in general $\boldsymbol{z} = (I - \Phi^\top)^{-1}\boldsymbol{q}$ is nonnegative if and only if $\boldsymbol{q}$ is nonnegative. This leaves how to incorporate mass balance relaxations (in order to take into account epistemic uncertainty) and other nonnegative terms under Gottschalk et al. [2010]'s parametrisation a possible direction for future work.

References

M. Betancourt. A Conceptual Introduction to Hamiltonian Monte Carlo. 2017. doi: 10.48550/ARXIV.1701.02434. URL https://arxiv.org/abs/1701.02434.

J. Dong, J. Liao, X. Huan, and D. Cooper. Expert elicitation and data noise learning for material flow analysis using bayesian inference. *Journal of Industrial Ecology*, 27(4):1105–1122, 2023. doi: 10.1111/jiec.13399.

A. Gelman, J. Carlin, H. Stern, D. Dunson, A. Vehtari, and D. Rubin. *Bayesian Data Analysis, Third Edition*. Chapman and Hall/CRC, 2013. ISBN 9781439840955.

F. Gottschalk, R. W. Scholz, and B. Nowack. Probabilistic material flow modeling for assessing the environmental exposure to compounds: Methodology and an application to engineered nano-TiO2 particles. *Environmental Modelling & Software*, 25(3):320–332, 2010. ISSN 1364-8152. doi: https://doi.org/10.1016/j.envsoft.2009.08.011. URL https://www.sciencedirect.com/science/article/pii/S1364815209002394.

T. E. Graedel, D. van Beers, M. Bertram, K. Fuse, R. B. Gordon, A. Gritsinin, E. M. Harper, A. Kapur, R. J. Klee, R. Lifset, L. Memon, and S. Spatari. The Multilevel Cycle of Anthropogenic Zinc. *Journal of Industrial Ecology*, 9(3):67–90, 2005. doi: https://doi.org/10.1162/1088198054821573. URL https://onlinelibrary.wiley.com/doi/abs/10.1162/1088198054821573.

T. Hastie, R. Tibshirani, and J. Friedman. *The Elements of Statistical Learning*. Springer Series in Statistics. Springer New York Inc., New York, NY, USA, 2001.

M. Hoffman and A. Gelman. The No-U-Turn Sampler: Adaptively Setting Path Lengths in Hamiltonian Monte Carlo. *Journal of Machine Learning Research*, 15:1953–1623, 2014.

R. A. Horn and C. R. Johnson. *Matrix Analysis*. Cambridge University Press, 1985.

R. C. Lupton and J. M. Allwood. Incremental Material Flow Analysis with Bayesian Inference. *Journal of Industrial Ecology*, 22(6):1352–1364, 2018. doi: https://doi.org/10.1111/jiec.12698. URL https://onlinelibrary.wiley.com/doi/abs/10.1111/jiec.12698.

M. Mannan and S. G. Al-Ghamdi. Environmental impact of water-use in buildings: Latest developments from a life-cycle assessment perspective. *Journal of Environmental Management*, 261:110198, 2020. ISSN 0301-4797. doi: https://doi.org/10.1016/j.jenvman.2020.110198. URL https://www.sciencedirect.com/science/article/pii/S030147972030133X.

L. Mirsky. A trace inequality of John von Neumann. *Monatshefte für Mathematik*, 79(4):303–306, Dec 1975. ISSN 1436-5081. doi: 10.1007/BF01647331. URL https://doi.org/10.1007/BF01647331.

A. W. v. d. Vaart. *Asymptotic Statistics*. Cambridge Series in Statistical and Probabilistic Mathematics. Cambridge University Press, 1998. doi: 10.1017/CBO9780511802256.
